# Surface-controlled reversal of the selectivity of halogen bonds

**DOI:** 10.1038/s41467-020-19379-4

**Published:** 2020-11-06

**Authors:** Jalmar Tschakert, Qigang Zhong, Daniel Martin-Jimenez, Jaime Carracedo-Cosme, Carlos Romero-Muñiz, Pascal Henkel, Tobias Schlöder, Sebastian Ahles, Doreen Mollenhauer, Hermann A. Wegner, Pablo Pou, Rubén Pérez, André Schirmeisen, Daniel Ebeling

**Affiliations:** 1 Institute of Applied Physics (IAP), Justus Liebig University Giessen, Heinrich-Buff-Ring 16, 35392 Giessen, Germany; 2 Center for Materials Research (LaMa), Justus Liebig University Giessen, Heinrich-Buff-Ring 16, 35392 Giessen, Germany; 3 Quasar Science Resources S.L., Camino de las Ceudas 2, E-28232 Las Rozas de Madrid, Spain; 4 Departamento de Física Teórica de la Materia Condensada, Universidad Autónoma de Madrid, E-28049 Madrid, Spain; 5 Institute of Physical Chemistry, Justus Liebig University Giessen, Heinrich-Buff-Ring 17, 35392 Giessen, Germany; 6 Institute of Organic Chemistry, Justus Liebig University Giessen, Heinrich-Buff-Ring 17, 35392 Giessen, Germany; 7 Condensed Matter Physics Center (IFIMAC), Universidad Autónoma de Madrid, E-28049 Madrid, Spain; 8 Present Address: Department of Physical, Chemical and Natural Systems, Universidad Pablo de Olavide, Ctra. Utrera Km. 1, E-41013 Seville, Spain; 9 Present Address: Institute of Nanotechnology, Karlsruhe Institute of Technology (KIT), Hermann-von-Helmholtz-Platz 1, 76344 Eggenstein-Leopoldshafen, Germany

**Keywords:** Chemistry, Physics

## Abstract

Intermolecular halogen bonds are ideally suited for designing new molecular assemblies because of their strong directionality and the possibility of tuning the interactions by using different types of halogens or molecular moieties. Due to these unique properties of the halogen bonds, numerous areas of application have recently been identified and are still emerging. Here, we present an approach for controlling the 2D self-assembly process of organic molecules by adsorption to reactive vs. inert metal surfaces. Therewith, the order of halogen bond strengths that is known from gas phase or liquids can be reversed. Our approach relies on adjusting the molecular charge distribution, i.e., the σ-hole, by molecule-substrate interactions. The polarizability of the halogen and the reactiveness of the metal substrate are serving as control parameters. Our results establish the surface as a control knob for tuning molecular assemblies by reversing the selectivity of bonding sites, which is interesting for future applications.

## Abstract (web summary)

Halogen bonds are emerging in numerous areas of application for building molecular assemblies due to their directionality and tunability. Here, the authors establish the surface as a control knob for tuning molecular assemblies by reversing the selectivity of bonding sites.

## Introduction

Intermolecular halogen bonds are ideally suited for controlling molecular self-assembly and designing new materials due to their strong directionality and tunability. With this unique combination of properties halogen bonds enable molecular assemblies with manifold conformations. This is particularly interesting for broad applications in the fields of supramolecular chemistry^1–4^, crystal engineering^5–7^, catalysis^8^, and drug design^9,10^. This potential was gradually realized during the last two decades when the nature of the halogen bond was better understood.

**Fig. 1:**
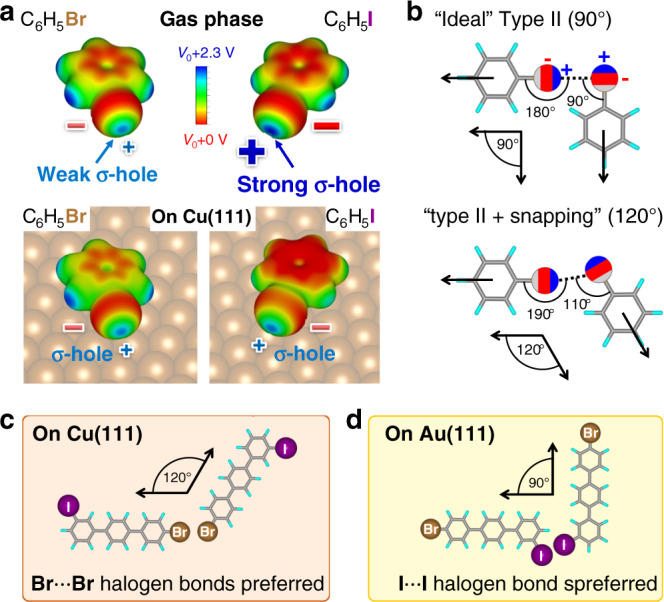
Charge distribution of halogen benzenes, scheme of type II halogen bonds, and bonding configurations on Cu(111) and Au(111). **a** Electrostatic potential (ESP) at the 10^−3^ e bohr^−3^ charge density isosurface as calculated by DFT of bromobenzene (C_6_H_5_Br) and iodobenzene (C_6_H_5_I) in gas phase and on Cu(111). The contribution of the substrate has been removed (see “Methods”). The “negative belts” and the “σ-holes” appear as red and blue regions, respectively (indicated by red “−” and blue “+” signs). **b** Schematic drawings of type II halogen bonds with effective bonding angles of 90° (upper part) and 120° (lower part), respectively. The effective bonding angles (*θ*_eff_ = −180° + *θ*_1_ + *θ*_2_) are measured between the *C*–*X* axes as indicated by black arrows. Black dotted lines between the positive σ-hole (blue region) and the negative belt (red region) of neighboring halogens are indicating the halogen bonds. **c**, **d** Sketch of observed molecular assemblies on the reactive Cu(111) and the inert Au(111) surface indicating the control over the bonding configuration and the selectivity.

Today, we know that the charge distribution of a halogen (X) that is covalently bound to an organic molecule (R) is anisotropic. Typically, a “belt” of high electron density is observed around the X–R bond axis, while the electron density at the cap of the halogen is significantly lower (see Fig. 1a). This region, the so-called “σ-hole”, can be even positive depending on the type of halogen and the organic rest^11^.

The σ-hole is accountable for both the directionality of the halogen bond and its tunability. As sketched in Fig. 1b for a C_6_H_5_–X⋯X–C_6_H_5_ contact (we indicate the non-covalent intermolecular halogen bond in the text with three dots and in the figures with dotted lines) the anisotropic charge distribution at the halogen favors an effective bonding angle of 90° (see upper part), however, slightly different angles of, e.g., 120° are also possible (see lower part). Both cases are typically classified in the literature as type II halogen bonds^12^ and in agreement with the actual IUPAC definition of the halogen bond^13^. So-called “head-on” configurations with an effective bonding angle of, or close to, 180° (type I halogen bonds) are only observed in densely packed crystals since these contacts do not arise from attractive electrostatic interactions.

Intermolecular halogen bonds can be tuned by either changing the type of halogen or the rest of the molecule. The strength of the σ-hole increases with the polarizability and decreases with the electronegativity of the halogen. Hence, in gas phase it follows the order I > Br > Cl > F^14,15^. This behavior is exemplarily shown in Fig. 1a for bromobenzene (C_6_H_5_Br) and iodobenzene (C_6_H_5_I) (see upper part). The σ-hole can also be effectively tuned by changing the molecular substituent that is bound to the halogen. In general, a substituent with high electronegativity will strengthen the σ-hole^15,16^. Therefore, e.g., the hybridization of the carbon atom that binds to the halogen plays a significant role.

Effects of different types of halogens have recently been revealed for molecular assemblies on surfaces. Using low temperature scanning probe microscopy with CO-functionalized tips^17,18^ it was demonstrated that even fluorinated compounds, which have an anisotropic charge distribution but do not develop a positive σ-hole due to the low polarizability of fluorine, form molecular clusters that are very similar to corresponding brominated compounds^19–21^. Interestingly, for brominated compounds, halogen bonded 2D molecular islands or networks with threefold symmetry have been observed on different surface materials, including Cu(111), Ag(111), and Au(111)^22–25^. Halogen-bonded assemblies with tetragonal packing, however, have only been reported for relative inert substrates such as Ag(111) or Au(111)^26–28^, which suggests that the reactivity of the surface material influences the halogen bonds.

Understanding the influence of the surface on the halogen bonds can provide alternative strategies for tuning halogen bonds and will be useful for future applications in different fields. In the field of 2D crystal engineering such knowledge can be applied for designing molecular networks with desired structures and properties^7^. The catalysis of organic reactions can also benefit from this knowledge, since the strength of the halogen bond is determining its catalytic activity^8^. Such information is also relevant to the field of on-surface chemistry. For example, it has been shown in the literature that on-surface coupling reactions can be directed using molecular self-assembly for designing novel compounds and structures^29,30^. Furthermore, some on-surface reactions, such as the Ullmann-type coupling involve halogenated precursors. Information about the influence of the surface on the halogen bonds can help to better understand the pathways and mechanisms of such on-surface reactions, what is important for designing new materials with unique properties^31–33^.

Here, we present an approach for tuning the strength and the directionality of halogen bonds by employing molecule–substrate interactions. As depicted in Fig. 1a, the charge distributions of bromobenzene and iodobenzene change significantly after adsorption to a Cu(111) surface. Since this effect is much stronger for the iodobenzene than for the bromobenzene, this will undoubtedly influence the relative strength of the σ-holes. Therefore, adsorption to a relative reactive surface, such as Cu(111) will strongly influence the molecular assemblies and can even reverse the selectivity of bonding sites. On an inert surface, such as Au(111), however, the gas phase order of the strength the σ-holes is practically retained. This opens the possibility of controlling the self-assembly process by changing the surface material or the type of halogen (see sketches in Fig. 1c, d). With our approach, we gain understanding about the selectivity of halogen bonds in dependence of the substrate material and its effect on the bonding geometry, which was missing until now.

## Results and discussion

### Selectivity of halogen bonds on Cu(111) and on Au(111)

**Fig. 2:**
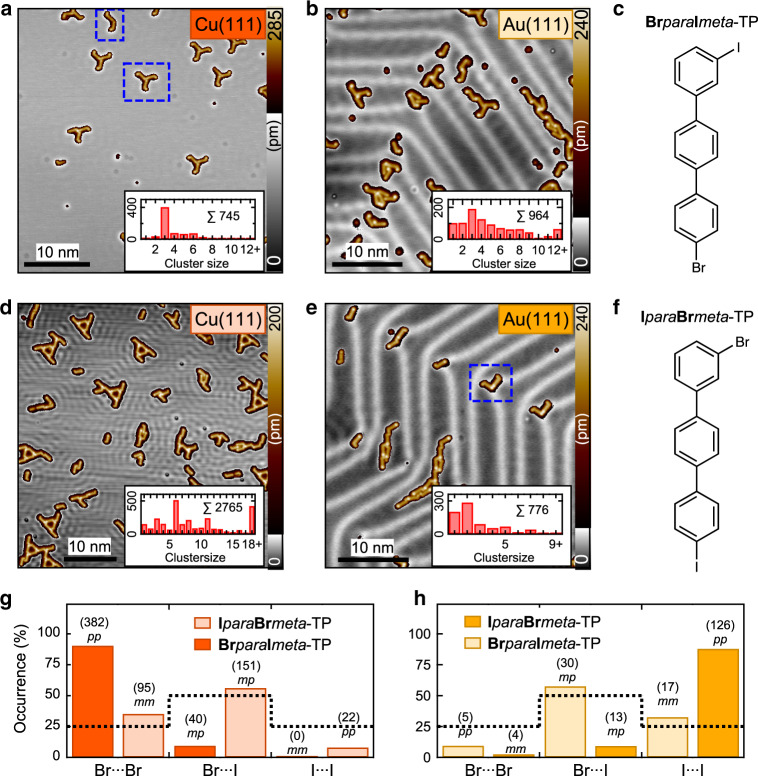
Overviews of halogen bonded clusters and selectivity of halogen bonds. **a**, **b**, **d**, **e** STM overview scans of **Br***para***I***meta*-TP and **I***para***Br***meta*-TP on Cu(111) and Au(111). The insets give the total numbers of counted molecules that were found in clusters of a certain size. The dashed blue boxes indicate typical windmills and dimers as analyzed in Fig. 3. **c**, **f** Chemical structures of **Br***para***I***meta*-TP (used in **a**, **b**) and **I***para***Br***meta*-TP (used in **d**, **e**). **g**, **h** Statistical distribution of Br⋯Br, I⋯I, and Br⋯I halogen bonds on Cu(111) and Au(111), respectively. On Cu(111) only dimers and trimers and on Au(111) only dimers are analyzed. The total number of counted halogen bonds are given in parentheses. *Para–para*, *para–meta*, or *meta–meta* connections are indicated by italic letters. The dashed black lines represent the statistical 25:50:25% distribution that would be observed if Br⋯Br, Br⋯I, and I⋯I connections were equal in strength. Parameters: sample bias voltage *U* = 100 mV, tunneling current *I* = 10 pA (**a**, **b**, **e**) and *I* = 100 pA (**d**).

**Fig. 3:**
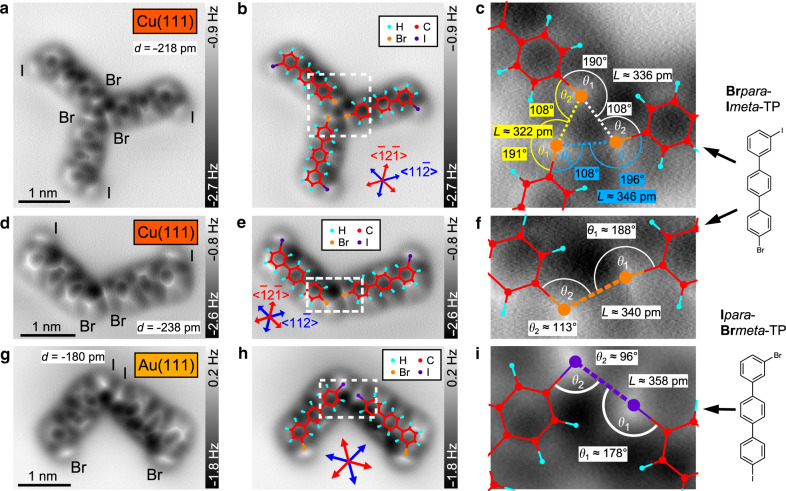
Bonding configurations of typical windmills and dimers on Cu(111) and Au(111). **a**–**c** AFM constant-height images of a Br···Br···Br windmill structure of **Br***para***I***meta*-TP on Cu(111). **b** AFM image overlaid with structural models of **Br***para***I***meta*-TP. Crystallographic [$\bar 12\bar 1$] and [$11\bar 2$] directions of the Cu(111) lattice are indicated with red and blue arrows, respectively. **c** Zoom-in to central region of the cluster with bonding angles *θ*_1_ and *θ*_2_ and bond distance *L* of the halogen bonds. Each bromine is acting as a halogen bond donor and acceptor, respectively (see Fig. 1b). **d**–**f** AFM image and bonding configuration of a Br···Br dimer [**Br***para***I***meta*-TP on Cu(111)]. **g**–**i** AFM image and bonding configuration of a I···I dimer [**I***para***Br***meta*-TP on Au(111)]. Parameters: oscillation amplitude 160 pm (**a**, **d**) and 170 pm (**g**), imaging distances are given with respect to the tunneling gap at *U* = 200 mV (**a**, **d**) and 100 mV (**g**) and *I* = 10 pA.

All scanning tunneling microscope (STM) and AFM images presented in this work have been measured at 5.2 K with a low-temperature STM/AFM (ScientaOmicron) and CO-functionalized metal tips (see “Methods”). The two structural isomers 4-bromo-3″-iodo-*p*-terphenyl (**Br***para***I***meta*-TP, see Fig. 2c) and 3-bromo-4″-iodo-*p*-terphenyl (**I***para***Br***meta*-TP, Fig. 2f) have been specifically designed for this study to serve as model compounds. By attaching the bromine and iodine atoms to the *para* and *meta* position (see Fig. 2c, f) of the terphenyl backbone the two halogens are electronically decoupled by an additional phenyl ring and an unambiguous identification of molecules is possible from large area STM scans^32^. The latter is beneficial for obtaining reliable statistics. Furthermore, by studying both isomers effects that arise from *para–para* or *meta–meta* type connections can be evaluated. The molecules were evaporated to precooled substrates to avoid dehalogenation. Low coverages were used to obtain small cluster sizes.

Figure 2a, b, d, e shows exemplary overview STM images of the two isomers **Br***para***I***meta*-TP and **I***para***Br***meta*-TP on Cu(111) and Au(111) and statistical information about the distribution of cluster sizes (dimers, trimers, tetramers, etc.). Therefore, the total number of molecules that were found in clusters with a certain size are given in the insets. Several of these STM images were used for determining the selectivity of the halogen bonds (Fig. 2g, h). The two different substrates Cu(111) and Au(111) have been chosen as model systems for a relatively reactive and a rather inert surface, respectively. Accordingly, the Cu(111) surface should show a strong influence on the selectivity, while the selectivity that is known from gas phase should be retained on Au(111).

To ensure that influences of the surface reconstruction on Au(111) or the requirements for building 2D networks on Cu(111) are excluded, we counted only the smallest clusters. This is essential, since on Cu(111), clusters with four or more molecules tend to rearrange into 2D Sierpinski networks (see Fig. S1)^25^. On Au(111), molecular trimers are already influenced by the herringbone reconstruction. Larger clusters form braid-like structures in the fcc regions of the herringbone reconstruction (Fig. 2b, e).

On Cu(111), Br⋯Br connections are strongly preferred over I⋯I connections for both studied isomers (Fig. 2g), which is completely unexpected since it is well known that iodobenzene has a stronger σ-hole than bromobenzene in the gas phase (see Fig. 1a). *Para–para* connections are favored over *meta–meta* connections. In the case of **Br***para***I***meta*-TP, we count 90.5% Br⋯Br (*para-para*), 0.0% I⋯I (*meta–meta*), and 9.5% Br⋯I (*para–meta*) connections (see dark orange bars). For **I***para***Br***meta*-TP, we observe 35.4% Br⋯Br (*meta–meta*), 8.2% I⋯I (*para-para*), and 56.3% Br⋯I (*meta–para*) connections (see bright orange bars).

By repeating these measurements on Au(111), we observe a reversed selectivity of the binding sites (Fig. 2h). Here, we count 88.0% I⋯I (*para–para*), 2.7% Br⋯Br (*meta–meta*), and 9.3% Br⋯I (*meta–para*) connections in case of **I***para***Br***meta*-TP (see dark yellow bars) and 32.7% I⋯I (*meta–meta*), 9.6% Br⋯Br (*para–para*), and 57.7% Br⋯I (*para–meta*) connections in case of **Br***para***I***meta*-TP (see bright yellow bars). Due to this surface induced reversal of the selectivity, the two graphs in Fig. 2g, h almost appear as mirror images of each other, which represents a remarkable control over the molecular assemblies. Please note that the reversal of bonding preference does not apply to larger clusters due to the mentioned influence of the herringbone reconstruction on Au(111) and the 2D network formation on Cu(111) (see Fig. S2).

### Bonding configurations on Cu(111) and Au(111)

The STM overview scans in Fig. 2a, d reveal that structures with effective bonding angles of 120° are predominant on Cu(111) (see dashed blue boxes). On Au(111), however, effective bonding angles close to 90° are observed, in particular, for molecular dimers (see dashed blue box in Fig. 2e). Next, we perform a detailed analysis of the bonding configuration and the adsorption conformations of the most abundant clusters using high resolution constant-height AFM images with CO-functionalized tips.

In Fig. 3a–f, we show a typical molecular trimer (so-called windmill) and a dimer of **Br***para***I***meta*-TP on Cu(111). Both structures are formed by Br⋯Br (*para–para*) connections. In Fig. 3g–i, a typical example for an I⋯I (*para–para*) connection of **I***para***Br***meta*-TP on Au(111) is depicted. By fitting the corresponding molecular structures to the AFM images (Fig. 3b, e, h) the bonding angles and distances can be precisely determined (see zoom-ins in Fig. 3c, f, i)^31^. Our findings can be summarized as follows: On Cu(111), bonding angles of *θ*_1_ ≈ 190° and *θ*_2_ ≈ 110° and bond lengths of 320–410 pm are observed for typical windmills and dimers (see Figs. 3a–f and S3). The molecular connections can be classified as type II halogen bonds with an effective bonding angle of *θ*_eff_ ≈ 120° (cf. Fig. 1b lower part). Approximately, 95% of the molecules, align with the [$11\bar 2$] directions (see Fig. S4), which is in agreement with previous findings for single adsorbed **Br***para***I***meta*-TP molecules on Cu(111)^32^. This indicates a strong snapping to the threefold Cu(111) surface lattice due to strong molecule–substrate interactions.

On Au(111), the measured bonding angles for the **I***para***Br***meta*-TP dimer are *θ*_1_ ≈ 178° and *θ*_2_ ≈ 96° (Fig. 3i). This results in an effective bonding angle of *θ*_eff_ ≈ 94° for the I⋯I (*para-para*) connection, which is very close to an ideal type II halogen bond (Fig. 1b). On Au(111), the snapping of the molecules with the [$11\bar 2$] directions is much weaker than on Cu(111) due to the lower molecule–substrate interactions. On Au(111), only 70% of the single molecules and only 55% of the molecules within dimers align with the [$11\bar 2$] directions (see Fig. S4). The latter number can be rationalized from Fig. 3h, which reveals that the left molecule aligns with the [$11\bar 2$] direction, while the right molecule is rotated by approximately 30°. This indicates that the snapping to the Au(111) lattice is overruled by the I⋯I halogen bond that favors an effective bonding angle of 90°.

Apparently, we also found one molecular dimer on Au(111) with an effective bonding angle close to 120° (see Fig. S5). However, this was one of the rare Br∙∙∙Br dimers of **I***para***Br***meta*-TP on Au(111) with a *meta–meta* type connection (≈2.7%, see Fig. 2e). For this particular case, the observed angle of 120° is presumably caused by the relatively weak Br∙∙∙Br halogen bond that is not able to overcome the snapping to the Au(111) lattice. These results highlight the strong influence of the surface material on the directionality of the halogen bonds and the bonding symmetry of the observed clusters.

### Adsorption positions, conformations, and charge distributions

**Fig. 4:**
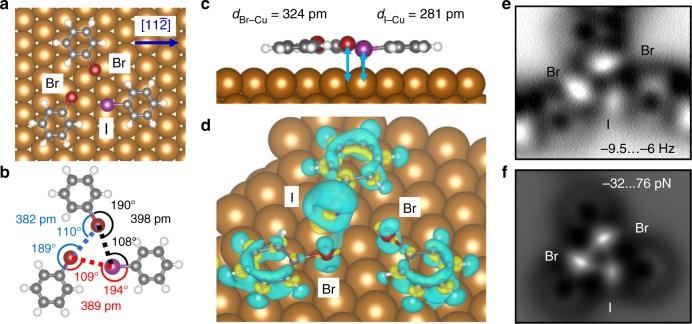
DFT calculated structure and charge transfer of a halobenzene Br∙∙∙Br∙∙∙I windmill on Cu(111). **a** Top view of DFT calculated windmill structure consisting of two bromobenzenes and one iodobenzene on Cu(111) revealing the adsorption positions of the molecules. The $\left[ {11\bar 2} \right]$ direction is indicated by the blue arrow. **b** Bonding angles and distances of structure in (**a**). The effective bonding angles of the three halogen bonds are 118°, 119°, and 123°, respectively. **c** Side view of (**a**). Blue arrows indicate the distance between the halogens and the Cu(111) surface plane. **d** Differential charge density at the 10^−4^ e bohr^−3^ isosurface (yellow = charge accumulation, blue = charge depletion). Due to the interaction with the Cu(111) surface, charge is depleted from the negative belts (blue regions) and accumulated at the σ-holes (yellow regions). For iodine this effect is much stronger than for bromine. **e** AFM constant height image of a Br···Br···I windmill structure of **I***para***Br***meta*-TP on Cu(111) (see details in Fig. S3). **f** Simulated force map for the structure in (**a**).

To identify the charge distribution of the different halogens on the surface and the formation energy of the molecular assemblies, we performed density functional theory (DFT) calculations for model systems of bromo- and iodobenzene on Cu(111). Figure 4 shows a Br∙∙∙Br∙∙∙I windmill that has been calculated by DFT using VASP^34^ with PBE + D3^35,36^, PAW pseudopotentials^37,38^, and a plane wave cutoff of 425 eV (see “Methods”). The adsorption positions, the conformations, and the bonding angles and distances of the three halobenzenes are in excellent agreement with our experimental findings.

More precisely, Fig. 4a reveals that the three halogen atoms are located close to Cu(111) bridge sites, the phenyl rings are located above fcc sites, and the X–C bond axes are aligned with the crystallographic $[11\bar 2]$ directions (see blue arrow). This is in agreement with our experimental findings for single **Br***para***I***meta*-TP and **I***para***Br***meta*-TP molecules on Cu(111) [Fig. S6 and ref. ^32^] and with theoretical findings for single halobenzenes [Fig. S7 and ref. ^39^]. Furthermore, the bonding angles and distances of the model system (Fig. 4b) match precisely with the values for a corresponding Br∙∙∙Br∙∙∙I windmill of **I***para***Br***meta*-TP on Cu(111) (Fig. S3). In addition, the side view in Fig. 4c reveals that the iodine is approximately 40 pm closer to the Cu(111) surface plane than the bromine.

The calculated adsorption conformation in Fig. 4c agrees with the AFM scans of single molecules [Fig. S6 and ref. ^32^] and molecular clusters (Figs. 3, 4e, and S3) on Cu(111), where the iodine atoms always appear significantly darker than the bromine atoms. Please note, on the inert Au(111) surface the iodine appears brighter than the bromine due to the almost planar adsorption conformation and the larger atomic radius of the iodine (Fig. S5)^40^. Since the adsorption conformation reflects the molecule–surface interactions, we also validated the image contrast by AFM simulations with a charge density based method^41–43^ that some of us recently introduced (Figs. 4f, S8, and S9). The simulated AFM images are in excellent agreement with the experimental scans (Fig. 4e, f), which confirms that the iodine is closer to the Cu(111) surface atoms than the bromine (see details in Figs. S8 and S9). Interestingly, this detailed analysis of the image contrast also reveals that the oval appearance of the halogens in the experimental AFM scans (Figs. 3, 4e, and S6) is a direct effect of the presence of the sigma hole, which creates an anisotropic electric field sensed by the AFM probe (see Figs. S8 and S9).

The lower iodine-Cu(111) distance clearly indicates a relatively strong interaction between the iodine and Cu(111) surface atoms, while the bromine–Cu interaction is relatively low. As shown in Fig. 4d, the molecule–substrate interactions cause a charge transfer that strongly affects the charge distribution at the halogen and thereby the halogen bonds. Depicted is the differential charge density at the 10^−4^ e bohr^−3^ isosurface, which has been calculated by subtracting the charge densities of the isolated substrate and the isolated windmill structure from the charge density of the adsorbed molecules inside the windmill on Cu(111). The yellow and blue regions indicate charge accumulation and depletion, respectively. The Cu(111) surface induces a charge depletion in the region of the negative belts (blue regions) and charge accumulation at the σ-holes (yellow regions), which results in a reduction of the strength of the halogen bonds at the surface for both halogens. Since this effect is much more pronounced for iodine than for bromine (Fig. 4d), we can rationalize the observed preference for Br⋯Br connections on Cu(111). Due to the higher polarizability of iodine, its σ-hole is weakened more strongly on the reactive Cu(111) surface, and consequently, its role in the cluster formation is downgraded. Interestingly, in the gas phase, the higher polarizability of iodine leads to a stronger σ-hole while on the surface the opposite is observed.

### Intermolecular energies of windmill structures

Table 1 gives a comparison of the intermolecular energies of different halobenzene windmill structures in gas phase and on the Cu(111) surface. In gas phase the Br∙∙∙Br∙∙∙I windmill is energetically more favorable than the Br∙∙∙Br∙∙∙Br structure by 18 meV. On the Cu(111) surface, however, the Br∙∙∙Br∙∙∙Br structure is favored by 30 meV. The on-surface intermolecular energies were calculated by subtracting the adsorption energy of the single molecules from the total formation energy of the windmill on Cu(111). In the next step it is important to determine whether this relevant energy difference is only caused by the observed charge transfer or if other possible energy contributions play a distinctive role. Indeed, the energy difference could also be produced by changes in the intermolecular interaction due to both global molecular displacements and intramolecular relaxations induced by the molecule–substrate interaction (for example, the vertical shift of the I atom towards the metal substrate could cost a relatively important energy, or by the variation on the adsorption energy of each molecule as, upon windmill formation, they are not in their optimal configuration for adsorption as an isolated molecule). We have calculated these two energy contributions, obtaining values of 7 meV for the Br∙∙∙Br∙∙∙I windmill (0 meV for the Br∙∙∙Br∙∙∙Br case) for the energy due to the relaxation and 6 meV (3 meV) for the former contribution (see “Methods” for details). None of these contributions can explain the observed reversal of intermolecular energies on the Cu(111) surface, leaving the surface induced charge redistribution and its effect on the σ-hole as the main possible explanation.

**Table 1:** Intermolecular energies of Br∙∙∙Br∙∙∙Br and Br∙∙∙Br∙∙∙I windmills of bromo- and iodobenzene in gas phase and on Cu(111).

Intermolecular energies (meV)	Br···Br···Br	Br···Br···I
Windmill in gas phase	−199	−217
Windmill on Cu(111)	−100	−70

See “Methods” for details about the calculation of formation energies.

Overall, the theoretical part of our study reveals a clear reduction of the formation energies of both types of windmill structures on the Cu(111) surface that are on the order of a hundred of meV (Table 1). This reduction is mainly caused by a charge redistribution between the σ-hole and the negative belt within the halogen atom as illustrated in Fig. 4d. This electronic effect is more pronounced for the iodine case, which results in lower formation energies for windmills that contain iodine. As revealed by our experimental analysis (see statistics in Fig. 2), this effect has a decisive influence on the 2D self-assembly process that is reflected by the different symmetries of the molecular assemblies and the reversed selectivity of the halogen bonds.

In summary, we present an approach for tuning 2D halogen bonded molecular assemblies via adsorption on relatively reactive vs. relatively inert model surfaces. An in-depth analysis of the assembly process is enabled using low temperature AFM with CO-functionalized tips and DFT calculations, which allows to precisely determine the adsorption positions and conformations of the molecules, the selectivity of the halogen bonds, the halogen bonding angles and distances, the charge distributions, and the intermolecular energies. This is essential for gaining insight into the tuning effect, since all these properties are influenced by molecule–substrate interactions. On the inert Au(111) surface, an almost planar adsorption conformation is observed in the AFM images, while on Cu(111) the iodine atoms shifts significantly toward the substrate plane due to strong halogen–substrate interactions. The observed clusters on Cu(111) and Au(111) substrates are formed via type II halogen bonds with effective bonding angles of 120° and 90°, respectively. On the Cu(111) surface Br⋯Br halogen bonds are strongly favored, while on Au(111) I⋯I halogen bonds are preferred. In particular, for *para–para* type connections the bond selectivity is on the order of 90%. The reversal of the bond selectivity is caused by halogen–substrate interactions that alter the charge distribution of the different halogens and lead to differences in the intermolecular energies. Therewith, we establish the surface material as a control knob for tuning the symmetry and the selectivity of halogen bonds, whereby the polarizability of the halogens and the reactiveness of the substrate material act as the main control parameters. These insights into the nature of halogen bonds represent an alternative approach for designing molecular assemblies. Furthermore, the conceptual idea that halogen bonds are tunable via reactive atoms in their close vicinity paves the way for future studies that address the general applicability of this phenomenon. This is appealing for applications of halogen bonds in different fields, such as crystal engineering, supramolecular chemistry, catalysis, drug design, or on-surface chemistry.

## Methods

### Synthesis of Br*para*I*meta*-TP and I*para*Br*meta*-TP

Details about the synthesis of **Br***para***I***meta*-TP and **I***para***Br***meta*-TP can be found in the Supplementary information. **Br***para***I***meta*-TP was synthesized according to the procedure reported in the literature^44^.

### Sample preparation

The Cu(111) and Au(111) crystals (MaTecK, Germany) were cleaned by multiple cycles of Ar+ sputtering (*E* = 1.5 kV, *I* = 3.6 µA, *p* = 6 × 10^−6^ mbar for initial cycles and *E* = 0.8 kV, *I* = 1.1 µA, *p* = 3 × 10^−6^ mbar for final cycles) and annealing (1000 K for initial cycles and 730 K for final cycles). The **Br***para***I***meta*-TP and **I***para***Br***meta*-TP molecules were evaporated on the surfaces by using a home-built evaporation device held at 100–150 °C^45^. The molecules were evaporated for several seconds onto the precooled metal substrates (*T* below 100 K) to avoid dehalogenation of the molecules.

### STM/AFM

STM and AFM images were obtained under UHV conditions (pressure below 1 × 10^−10^ mbar) at a temperature of 5.2 K with a low temperature AFM/STM system (Scienta Omicron, Germany). In our setup the tip is grounded while the sample is connected to the bias voltage. A qPlus tuning fork sensor with a tungsten tip was used^46^. The tip was sharpened by voltage pulses and indentations into the respective metal surface. The AFM tip was functionalized with a CO molecule using standard procedures described in the literature^47^. Therefore, on Cu(111) the tip was first positioned above a CO molecule with a sample bias of +2 V and a tunneling setpoint of 1 nA. Subsequently, the feedback was deactivated and the voltage was ramped from +3 to 0 V while the tip-substrate distance was simultaneously reduced by 100 pm. In case of Au(111), CO can be unintentionally picked up by scanning the surface or by voltage pulses. The tip functionalization with CO was confirmed by scanning other adsorbed CO molecules. The resonance frequencies and quality factors of the sensors were ranging from *f*_res_ ≈ 19 to 27 kHz and *Q* ≈ 10,000 to 30,000, respectively. The qPlus sensor was operated in frequency modulation mode with constant amplitude using an external phase-locked loop electronics (MFLI, Zürich Instruments, Switzerland) for obtaining both STM and AFM images. Oscillation amplitudes in the range of 40–170 pm were used.

### First-principles calculations and AFM theoretical images

First, we characterize our halogen-molecule system by means of DFT calculations. We used the VASP package^34^ with a cutoff energy of 425 eV for the plane-wave basis set. The Perdew–Burke–Ernzerhof (PBE) functional^35^ supplemented by Grimme’s D3 correction for dispersion interactions^36^ was used to describe the exchange and correlation electronic interactions. The projected augmented wave method^37,38^ was used to build the pseudopotentials. Convergence criteria for the electronic self-consistent loop and the conjugate gradient algorithm was set to 10^−5^ eV and 0.01 eV Å^−1^, respectively. Due to the large size of the **Br***para***I***meta*-TP and **I***para***Br***meta*-TP molecules we studied the halogen bonds of a system constituted by smaller bromobenzene and iodobenzene molecules deposited on a three-layer Cu(111) slab. Since interactions with the subsurface atoms are important for the adsorption conformation of the molecules, including several Cu(111) layers is crucial^40,45,48,49^. These halobenzene molecules adsorbed on a triangular arrangement with the halogen atoms on bridge positions and the benzene rings on fcc-hollow positions are suitable models to accurately reproduce the experimental observations.

In addition, we use our recently developed method to simulate high resolution AFM images^41–43^. This method is able to produce theoretical AFM images at different heights by constructing a potential using some inputs from previous DFT calculations, essentially the total charge densities of the tip and the sample, and the electrostatic potential of the sample. The method just requires fitting two parameters to get a proper description of the Pauli repulsion given by $V_{{\mathrm{SR}}} = V_0{\int} {\left[ {\rho ^{{\mathrm{tip}}}\left( {{\boldsymbol{r}},R_{{\mathrm{tip}}}} \right)\rho ^{{\mathrm{sam}}}\left( {\boldsymbol{r}} \right)} \right]^\alpha } d{\boldsymbol{r}}$. Here, they were set to $V_0 = 32.7\left[ {\rm{eV}} \right],\alpha = 1.11$. The relaxation of the CO probe was accounted through a tilt potential, $V_{{\mathrm{tilt}}} = \frac{1}{2}{k^\prime} ({\mathrm{{\Delta} }}x^2 + {\mathrm{{\Delta} }}y^2)$, where *k*′ was set to 0.46 J m^−2^. Notice that, following the approach introduced by Hapala et al.^50^, we have minimized the probe position approaching the CO-sample interaction for those given by a rigid tip and restraining it to the original plane.

Please find more information about the DFT calculations and other computational details in the Supplementary information.

## Supplementary information

Supplementary Information

## Data Availability

The data supporting the findings of this study are available within the paper and its Supplementary Information files.

## References

[1] Metrangolo P, Resnati G (2001). Halogen bonding: a paradigm in supramolecular chemistry. Chemistry.

[2] Gilday LC (2015). Halogen bonding in supramolecular chemistry. Chem. Rev..

[3] Zheng Q-N (2015). Formation of halogen bond-based 2D supramolecular assemblies by electric manipulation. J. Am. Chem. Soc..

[4] Niyas MA, Ramakrishnan R, Vijay V, Sebastian E, Hariharan M (2019). Anomalous halogen-halogen interaction assists radial chromophoric assembly. J. Am. Chem. Soc..

[5] Mukherjee A, Tothadi S, Desiraju GR (2014). Halogen bonds in crystal engineering: like hydrogen bonds yet different. Acc. Chem. Res..

[6] Albright E, Cann J, Decken A, Eisler S (2017). Halogen···halogen interactions in diiodo-xylenes. CrystEngComm.

[7] Mukherjee A (2019). Halogenated building blocks for 2D crystal engineering on solid surfaces: lessons from hydrogen bonding. Chem. Sci..

[8] Sutar RL, Huber SM (2019). Catalysis of organic reactions through halogen bonding. ACS Catal..

[9] Lu Y (2009). Halogen bonding—a novel interaction for rational drug design?. J. Med. Chem..

[10] Kolar M, Hobza P, Bronowska AK (2013). Plugging the explicit sigma-holes in molecular docking. Chem. Commun..

[11] Clark T, Hennemann M, Murray JS, Politzer P (2007). Halogen bonding: the sigma-hole. J. Mol. Model..

[12] Desiraju GR, Parthasarathy R (1989). The nature of halogen···halogen interactions: are short halogen contacts due to specific attractive forces or due to close packing of nonspherical atoms?. J. Am. Chem. Soc..

[13] Desiraju GR (2013). Definition of the halogen bond (IUPAC Recommendations 2013). Pure Appl. Chem..

[14] Desiraju GR, Harlow RL (1989). Cyano-halogen interactions and their role in the crystal-structures of the 4-halobenzonitriles. J. Am. Chem. Soc..

[15] Cavallo G (2016). The halogen bond. Chem. Rev..

[16] Riley KE (2011). Halogen bond tunability I: the effects of aromatic fluorine substitution on the strengths of halogen-bonding interactions involving chlorine, bromine, and iodine. J. Mol. Model..

[17] Gross L, Mohn F, Moll N, Liljeroth P, Meyer G (2009). The chemical structure of a molecule resolved by atomic force microscopy. Science.

[18] Chiang C-l, Xu C, Han Z, Ho W (2014). Real-space imaging of molecular structure and chemical bonding by single-molecule inelastic tunneling probe. Science.

[19] Kawai S (2015). Extended halogen bonding between fully fluorinated aromatic molecules. ACS Nano.

[20] Han Z (2017). Imaging the halogen bond in self-assembled halogenbenzenes on silver. Science.

[21] Gallardo A, Fanfrlík J, Hobza P, Jelínek P (2019). Nature of binding in planar halogen-benzene assemblies and their possible visualization in scanning probe microscopy. J. Phys. Chem. C.

[22] Strohmaier R, Ludwig C, Petersen J, Gompf B, Eisenmenger W (1994). Stm investigations of C6Br6 on Hopg and MoS2. Surf. Sci..

[23] Walch H, Gutzler R, Sirtl T, Eder G, Lackinger M (2010). Material- and orientation-dependent reactivity for heterogeneously catalyzed carbon-bromine bond homolysis. J. Phys. Chem. C.

[24] Wang W, Shi X, Wang S, Hove MAV, Lin N (2011). Single-molecule resolution of an organometallic intermediate in a surface-supported ullmann coupling reaction. J. Am. Chem. Soc..

[25] Shang J (2015). Assembling molecular Sierpinski triangle fractals. Nat. Chem..

[26] Chung K-H (2011). Polymorphic porous supramolecular networks mediated by halogen bonds on Ag(111). Chem. Commun..

[27] Yoon JK (2011). Visualizing halogen bonds in planar supramolecular systems. J. Phys. Chem. C.

[28] Huang H (2016). Competition between hexagonal and tetragonal hexabromobenzene packing on Au(111). ACS Nano.

[29] Zhang H (2016). Two-dimensional chirality transfer via on-surface reaction. J. Am. Chem. Soc..

[30] Li Q (2018). Self-assembly directed one-step synthesis of [4]radialene on Cu(100) surfaces. Nat. Commun..

[31] Zint S (2017). Imaging successive intermediate states of the on-surface Ullmann reaction on Cu(111): role of the metal coordination. ACS Nano.

[32] Ebeling D (2019). Adsorption structure of mono- and diradicals on a Cu(111) surface: chemoselective dehalogenation of 4-bromo-3′-iodo-. ACS Nano.

[33] Fan Q (2019). Nanoribbons with nonalternant topology from fusion of polyazulene: carbon allotropes beyond graphene. J. Am. Chem. Soc..

[34] Kresse G, Furthmüller J (1996). Efficient iterative schemes for ab initio total-energy calculations using a plane-wave basis set. Phys. Rev. B.

[35] Perdew JP, Burke K, Ernzerhof M (1996). Generalized gradient approximation made simple. Phys. Rev. Lett..

[36] Grimme S, Antony J, Ehrlich S, Krieg H (2010). A consistent and accurate ab initio parametrization of density functional dispersion correction (DFT-D) for the 94 elements H-Pu. J. Chem. Phys..

[37] Blöchl PE (1994). Projector augmented-wave method. Phys. Rev. B.

[38] Kresse G, Joubert D (1999). From ultrasoft pseudopotentials to the projector augmented-wave method. Phys. Rev. B.

[39] Björk J, Hanke F, Stafström S (2013). Mechanisms of halogen-based covalent self-assembly on metal surfaces. J. Am. Chem. Soc..

[40] Zhong Q (2018). Symmetry breakdown of 4,4′-diamino-p-terphenyl on a Cu(111) surface by lattice mismatch. Nat. Commun..

[41] Ellner M (2016). The electric field of co tips and its relevance for atomic force microscopy. Nano Lett.

[42] Ellner M, Pou P, Pérez R (2017). Atomic force microscopy contrast with CO functionalized tips in hydrogen-bonded molecular layers: Does the real tip charge distribution play a role?. Phys. Rev. B.

[43] Ellner M, Pou P, Pérez R (2019). Molecular identification, bond order discrimination, and apparent intermolecular features in atomic force microscopy studied with a charge density based method. ACS Nano.

[44] Ebeling D (2017). Chemical bond imaging using higher eigenmodes of tuning fork sensors in atomic force microscopy. Appl. Phys. Lett..

[45] Zint S, Ebeling D, Ahles S, Wegner HA, Schirmeisen A (2016). Subsurface-controlled angular rotation: triphenylene molecules on Au(111) substrates. J. Phys. Chem. C.

[46] Giessibl FJ (1998). High-speed force sensor for force microscopy and profilometry utilizing a quartz tuning fork. Appl. Phys. Lett..

[47] Bartels L, Meyer G, Rieder KH (1997). Controlled vertical manipulation of single CO molecules with the scanning tunneling microscope: a route to chemical contrast. Appl. Phys. Lett..

[48] Ohmann R, Levita G, Vitali L, De Vita A, Kern K (2011). Influence of subsurface layers on the adsorption of large organic molecules on close-packed metal surfaces. ACS Nano.

[49] Mollenhauer D, Gaston N, Voloshina E, Paulus B (2013). Interaction of pyridine derivatives with a gold (111) surface as a model for adsorption to large nanoparticles. J. Phys. Chem. C.

[50] Hapala P (2014). Mechanism of high-resolution STM/AFM imaging with functionalized tips. Phys. Rev. B.

