# Supplementary material for: Surface-controlled reversal of the selectivity of halogen bonds
Source: Nat Commun. 2020 Nov 6;11:5630. doi: 10.1038/s41467-020-19379-4 (PMC7648107; doi:10.1038/s41467-020-19379-4)
Supplement: Supplementary file 1 — Supplementary Information [file 41467_2020_19379_MOESM1_ESM.pdf]

Supplementary Information for

Surface-Controlled Reversal of the Selectivity of Halogen Bonds

Jalmar Tschakert[1,2], Qigang Zhong[1,2], Daniel Martin-Jimenez[1,2], Jaime Carracedo-Cosme[3,4], Carlos Romero-Muñiz[4]†, Pascal Henkel[2,5], Tobias Schlöder[2,5]‡, Sebastian Ahles[2,6], Doreen Mollenhauer[2,5], Hermann A. Wegner[2,6], Pablo Pou[4,7], Rubén Pérez[4,7], André Schirmeisen[1,2], and Daniel Ebeling[1,2]*

Correspondence to: Daniel.Ebeling@ap.physik.uni-giessen.de

This PDF file includes:

- Supplementary Figures S1 to S9 (pages 2 – 10)
- Further details of first-principles calculations and AFM theoretical images (pages 11 – 13)
- Synthesis of **Br***para***I***meta*-TP (pages 14 – 16)
- Synthesis of **I***para***Br***meta*-TP (pages 17 – 20)
- References (page 21)

Supplementary Figure S1 – Sierpinski triangles

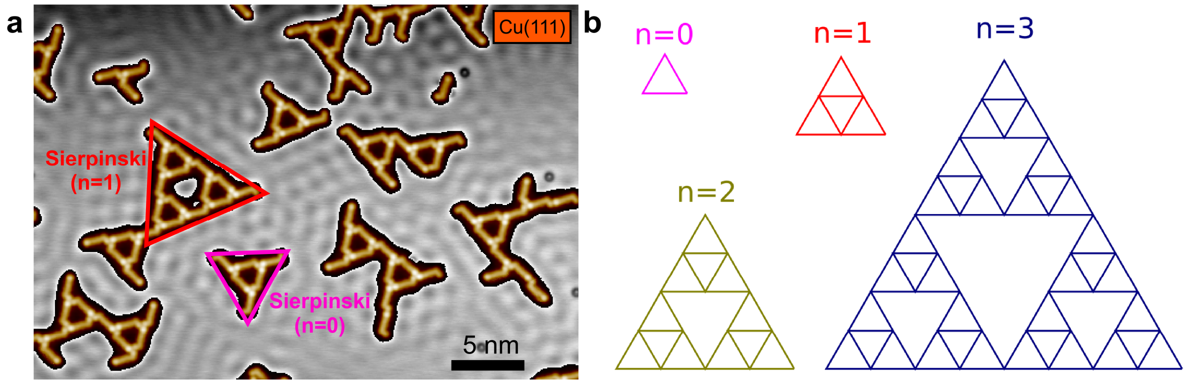

Figure S1. Sierpinski structures of 3-Bromo-4"-iodo-*p*-terphenyl (I*para*Br*meta*-TP) on Cu(111). (**a**) STM overview scans of **I***para***Br***meta*-TP on Cu(111). The molecular coverage in this image is slightly higher than in Figure 2d. Hence, larger clusters are formed. The larger triangular structures are composed of smaller triangular building blocks (see pink and red triangles) as in the so-called Sierpinski triangle fractal. (**b**) Schematic of Sierpinski structures for the orders $n = 0$ to 3.

Supplementary Figure S2 – Bond ratios for larger molecular clusters

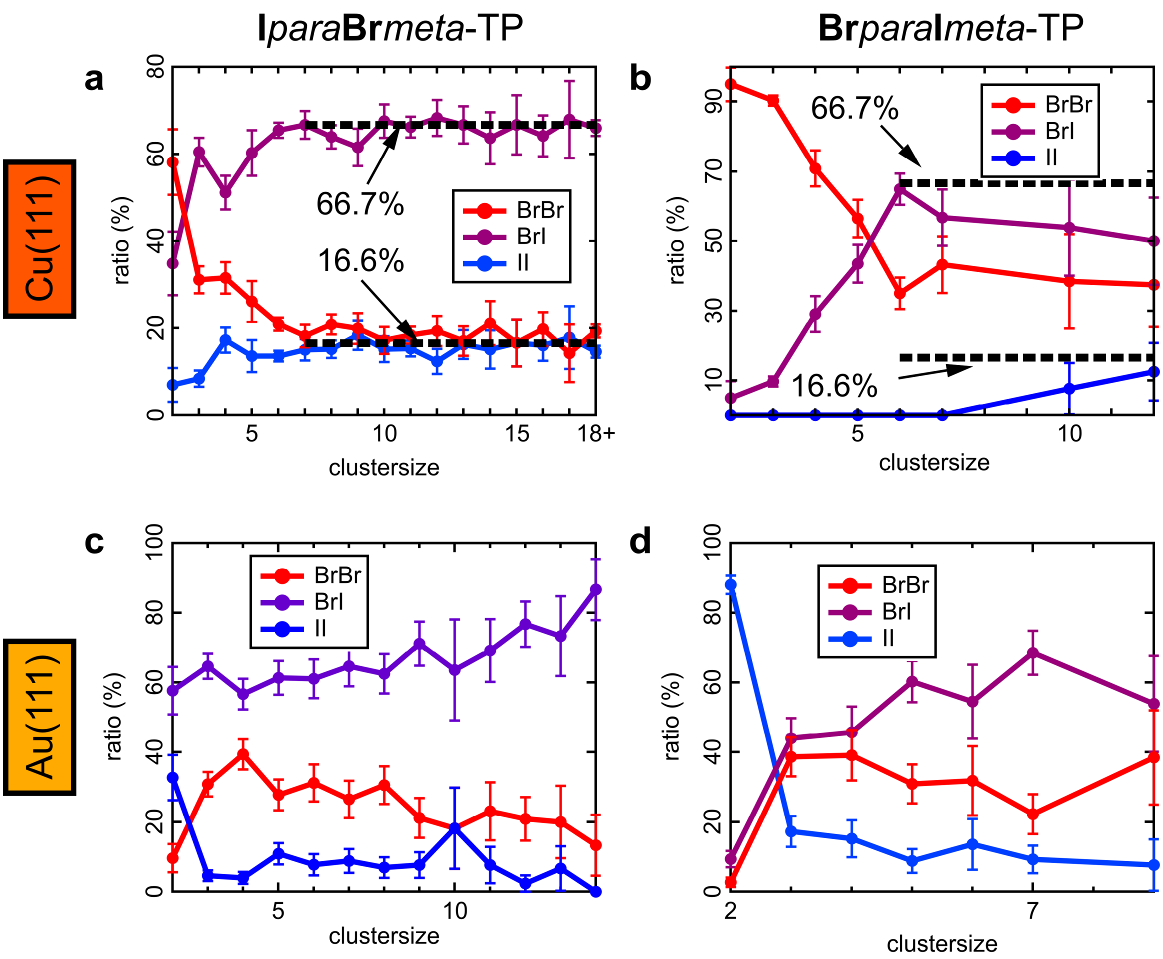

Figure S2. Ratio of type of halogen bonds (Br···Br *vs.* I···I *vs.* Br···I) as a function of the cluster size. (**a**,**b**) Bond rations for **Br***para***I***meta*-TP and **I***para***Br***meta*-TP on Cu(111). (**c**,**d**) Bond rations for **Br***para***I***meta*-TP and **I***para***Br***meta*-TP on Au(111). For the presented analysis of the halogen bonds in the manuscript only small clusters are taken into account [only dimers and trimers for Cu(111) and only dimers for Au(111)]. Larger clusters are excluded since 2D packing requirements and the surface reconstruction are clearly influencing those larger structures. The graph in (**a**), e.g., reveals, for clusters with a seven and more molecules, a constant ratio of 17% Br···Br, 17% I···I, and 67% Br···I bonds for **Br***para***I***meta*-TP on Cu(111). This ratio exactly matches the theoretical ratio of bonds that is needed for forming Sierpinski triangles (see Figure S1). On Au(111) the larger clusters form braid-like structures in the fcc regions of the herringbone reconstruction (Figure 2b,e), which also significantly influences the observed bond ratios.

Supplementary Figure S3 – Bonding configuration of a I*para*Br*meta*-TP "Br···Br···I" windmill on Cu(111). Direct comparison with theory.

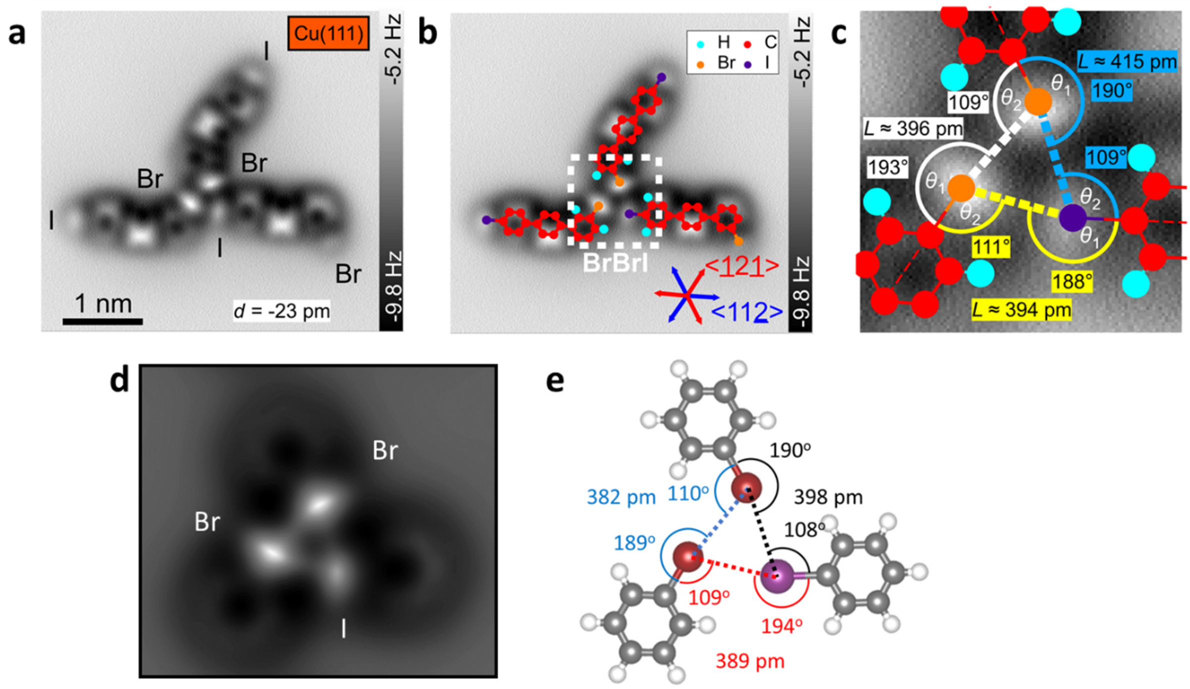

Figure S3. I*para*Br*meta*-TP windmill on Cu(111). (**a-c**) AFM constant-height images of a Br···Br···I windmill structure of **I***para***Br***meta*-TP on Cu(111) (same measurement as shown in Figure 4e). (**b**) AFM image with chemical structures of **I***para***Br***meta*-TP superimposed. Crystallographic $\langle\bar{1}2\bar{1}\rangle$ and $\langle 11\bar{2}\rangle$ directions of the Cu(111) lattice are indicated with red and blue arrows, respectively. (**c**) Zoom-in to central region of the cluster with bonding angles Θ_1 and Θ_2 and bond distance L for each of the three halogen bonds. The effective bonding angle is close to 120° for all three halogen bonds. (**d**,**e**) Simulated force map and bonding configuration of the calculated structure (same panel as in Figure 4f). The bonding angles and distances of the calculated structure (panel **e**) and the experimental AFM images (panel **c**) are in remarkable agreement. Parameters: (**a-c**) amplitude = 40 pm, imaging distance = -23 pm with respect to U = 100 mV and I = 100 pA.

Supplementary Figure S4 – Snapping of I*para*Br*meta*-TP monomers and dimers to substrate lattices

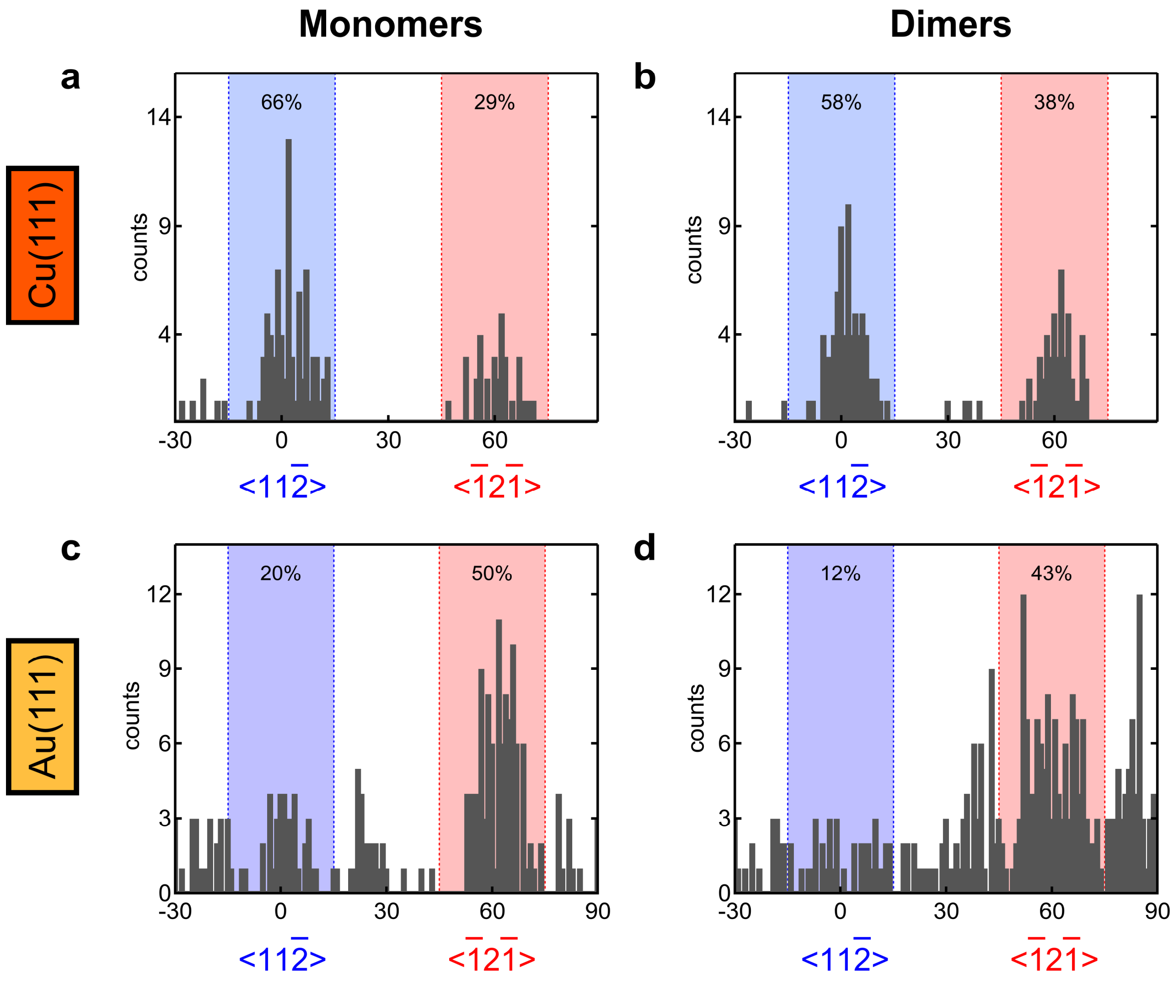

Supplementary Figure S4. Snapping of I*para*Br*meta*-TP monomers and dimers to the Cu(111) and Au(111) substrate lattices. Given are the counted number of molecules *vs.* their orientation on the substrate lattice for Cu(111) (**a**,**b**) and Au(111) (**c**,**d**). The orientation angles have been determined from several STM overview scans. Due to the uncertainty in the angle measurement a range of +- 15° has been chosen around [11-2] and [-12-1] directions, respectively (blue and pink regions). Molecules within this range are considered as "snapped to the lattice". For Cu(111) approximately 95 % of the molecules are found within this range, irrespectively if monomers (**a**) or dimers (**b**) are considered. On Au(111) a weaker snapping effect is observed. Only 70% of the single molecules (**c**) and only 55 % of the molecules within dimers (**d**) snap to the Au lattice.

Supplementary Figure S5 – 120° Dimer on Au(111)

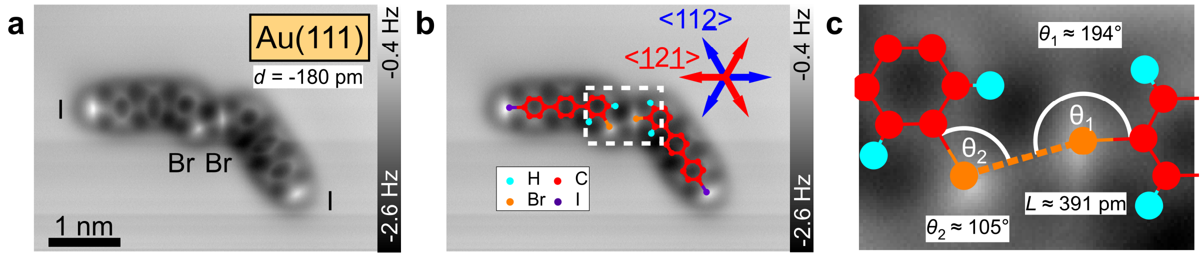

Figure S5. I*para***Br***meta*-TP **dimer on Au(111) with an effective bonding angle of 120°.** (**a**,**b**,**c**) Constant-height AFM images and zoom-in of a Br···Br type dimer with *meta-meta* connection of **I***para***Br***meta*-TP on Au(111). Br···Br connections of this compound are very seldom on Au(111) (occurrence of only 2.7%, see statistics in Figure 2h). Only a single dimer with this particular bonding configuration has been found in our study. The effective bonding angle is θ_{eff} = -180° + 194° + 105° = 119°.

Supplementary Figure S6 – Adsorption conformation of I*para*Br*meta*-TP on Cu(111)

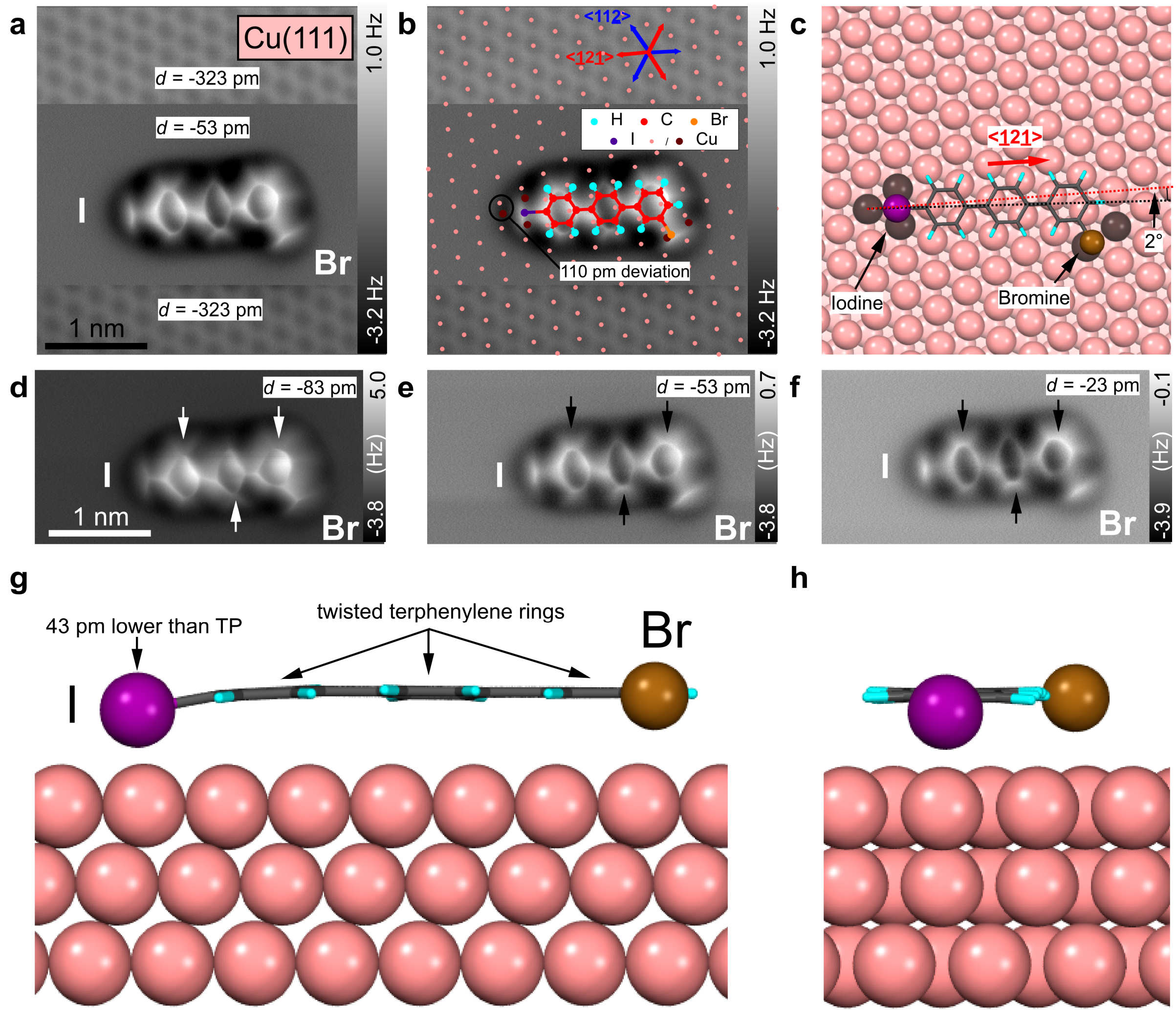

Figure S6. Adsorption conformation of single I*para*Br*meta*-TP on Cu(111). (**a**,**b**) AFM constant-height images of a single I*para*Br*meta*-TP on Cu(111). Two different imaging distances have been chosen in order to resolve the substrate atoms in the lower and upper parts of the image and the molecule in the center. Copper top sites are indicated in (**b**) by pink dots. (**c**,**g**,**h**) DFT calculated adsorption structure of I*para*Br*meta*-TP on Cu(111) [cf. Ref. [1]]. The adsorption position of the fitted structure in (**b**) is in good agreement with the calculated structure in (**c**), except of the [-12-1] directions, which are pointing in opposite directions. (**d**,**e**,**f**) Three constant-height AFM images of the molecule in (**a**) for three different imaging distances, which reveal the twisting angles of the three phenyl rings qualitatively. These twisting angles are in good agreement with the calculated structure (see **g** and **h** and Ref. [1]). Furthermore, the image contrast agrees well with the simulated AFM images of I*para*Br*meta*-TP (see Figure S9). The iodine atom is located 43 pm closer to the Cu(111) surface than the bromine atom (see panel **g**) due to stronger iodine-Cu interactions.

Supplementary Figure S7 – DFT structures of single bromobenzene and iodobenzene on Cu(111)

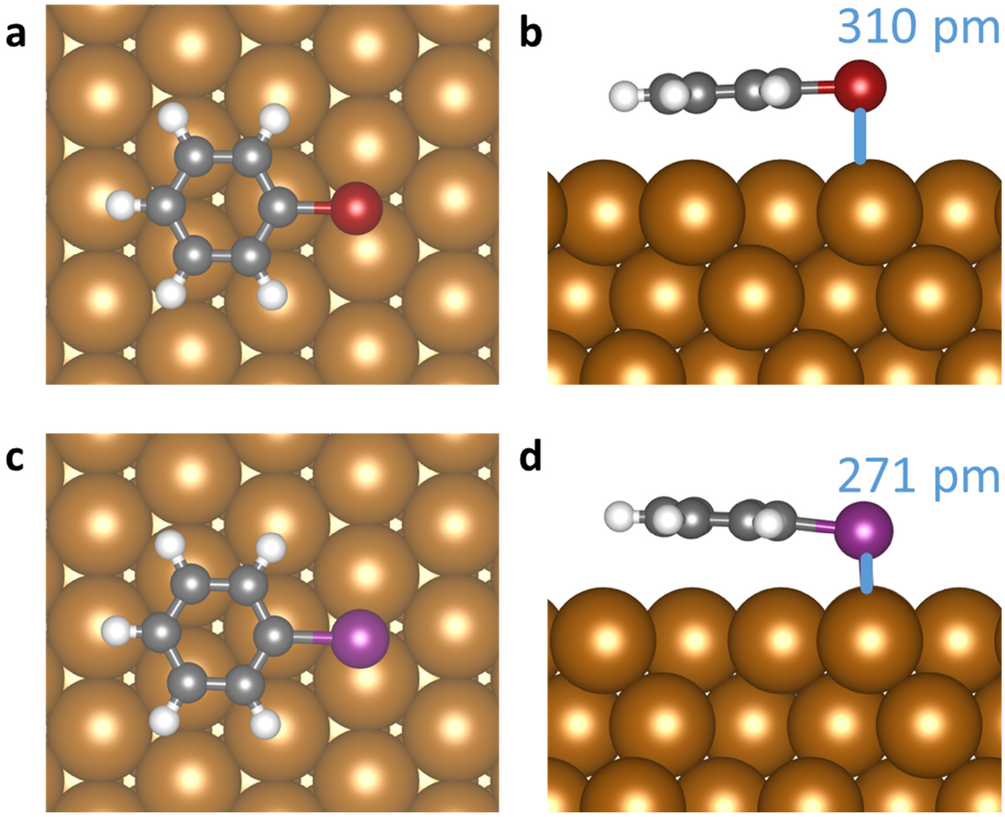

Supplementary Figure S7. Calculated adsorption structures [DFT Method] of single bromobenzene and iodobenzene molecules on Cu(111). (**a**,**b**) Top and side views of bromobenzene on Cu(111). (**c**,**d**) Top and side views of iodobenzene on Cu(111). The adsorption positions of the single molecules are very similar to the adsorption positions of molecules within windmill structures (Figure 4) or single **Br***para***I***meta*-TP and **I***para***Br***meta*-TP molecules [Figure S6 and Ref. [1]]. The halogens are located close to bridge sites, the phenyl rings are located above fcc sites, and the X-C bond is aligned with the crystallographic [11-2] direction. The side views (**b**,**d**) reveal that the iodine is approximately 40 pm closer to the Cu(111) surface plane than the bromine.

Supplementary Figure S8 – Contrast of simulated AFM images for windmill structures on Cu(111)

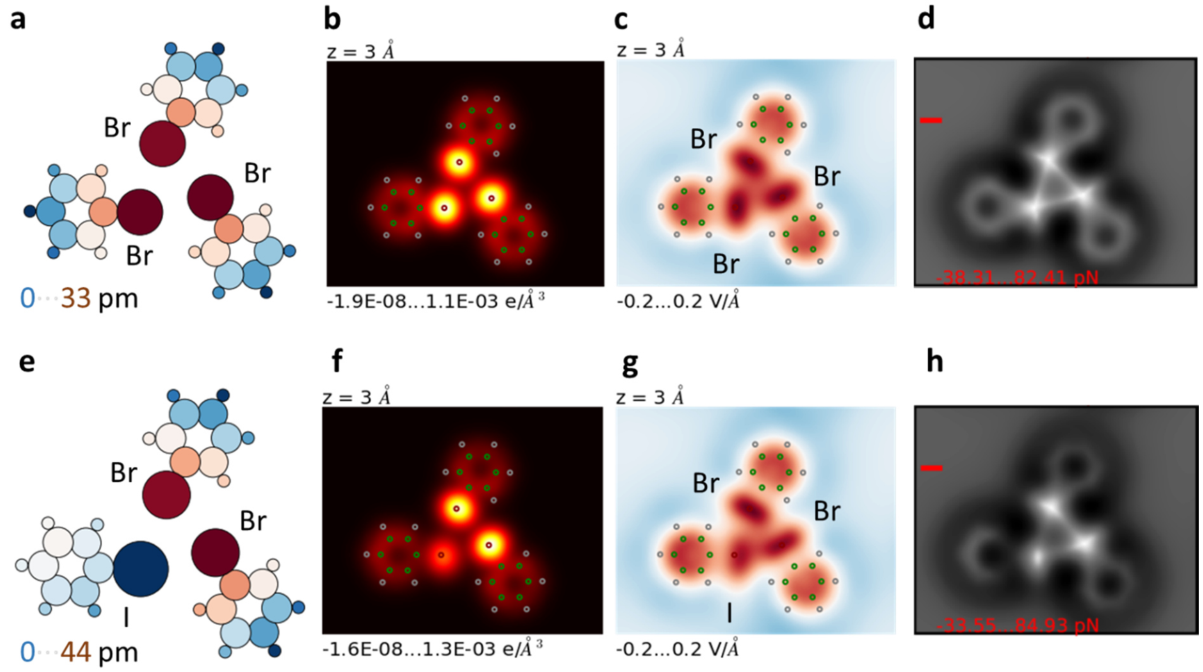

Figure S8. Contrast origin of the AFM images. (**a**,**e**) Atomic height color maps, (**b**,**f**) charge densities, (**c**,**g**) electrostatic potentials at 3 Å imaging distance, and (**d**,**h**) simulated force maps at 3.51 Å imaging distance for the Br···Br···Br (**a-d**) and Br···Br···I (**e-h**) windmill structures on the Cu(111) surface as calculated by DFT and our HR-AFM method. The atomic height map in panel (**e**) clearly shows that the iodine atom is downshifted towards the Cu(111) surface, which leads to lower Pauli repulsion between the CO-tip and the iodine atom. The charge densities in (**b**,**f**) reveal the characteristic large electron density around the halogens (yellow regions), which is higher for the bromine atoms than for the iodine atoms. Since the CO-tip has a slight negative charge at its apex, a lower repulsive electrostatic force for the iodine is found compared to bromine atoms. Both effects, i.e. the lower electrostatic repulsion and the lower Pauli repulsion lead to a less bright feature for the iodine atoms in the HR-AFM image (see panel **h**). The oval shape of the charge density at the halogens is difficult to discern from the images in (**b**,**f**). However, the electrostatic potential shown in (**c**,**g**) clearly reveals negative oval shaped areas at the halogen atoms (red regions). These repulsive regions are in concordance with the "negative belts" around the halogens (see Figure 1a). The surface potential at the caps of the halogens (at the σ-holes) is less negative (less repulsive). The relaxation of the probe enhances the asymmetry introduced by the electrostatic potential resulting in oval shaped features in the HR-AFM images (**d**,**h**) at the halogens. These oval contrast features are a direct fingerprint of the σ-holes of the halogens.

Supplementary Figure S9 – Contrast of simulated AFM images for I*para*Br*meta*-TP on Cu(111)

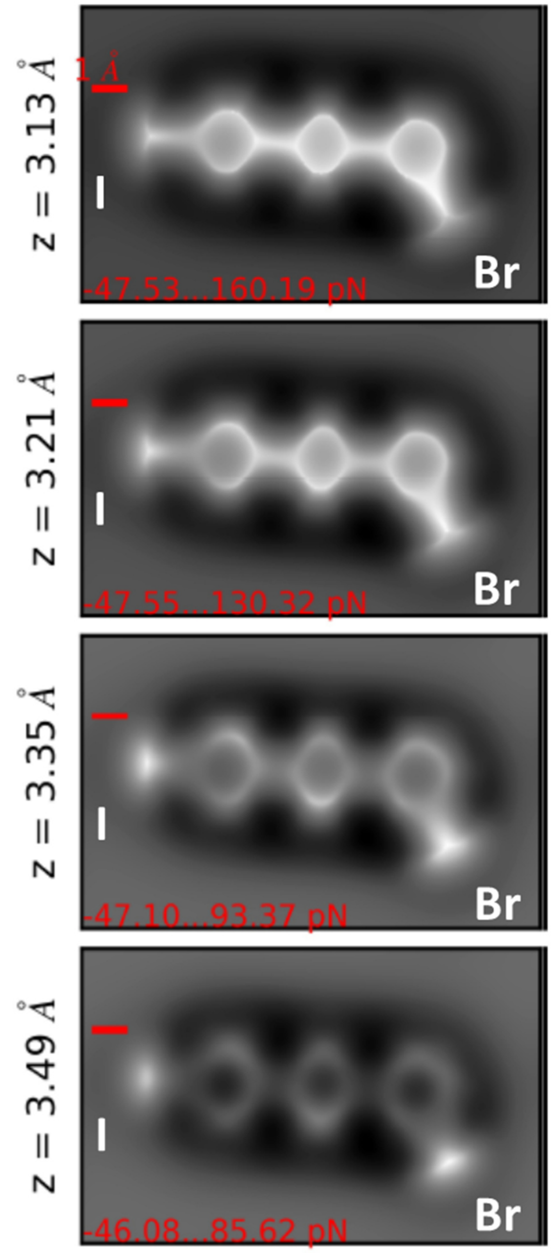

Figure S9. Contrast of simulated AFM images for I*para*Br*meta*-TP on Cu(111). The four panels show simulated force maps at four different imaging distances. The contrast features in these images are very similar to the features observed in experimental AFM scans [see Figure S6 and Ref. [1] for **Br***para***I***meta*-TP on Cu(111)]. The simulated images clearly reproduce the observed twisting angles of the phenyl rings, the different brightness of iodine and bromine (iodine appears darker due to the smaller I-Cu distance and lower electrostatic repulsion), and the oval features of the halogens (see explanation in Figure S8). Furthermore, as a consequence of the flexibility of the CO-tip the features change with decreasing tip-sample distance. This is also well reproduced by the simulated images. For example, the halogens appear at large distances as oval features. At smaller distances these features turn into sharp lines, which is in remarkable agreement with our experimental scans [see Figure S6d-f for **I***para***Br***meta*-TP on Cu(111)].

Further details of first-principles calculations and AFM theoretical images

To reproduce the experimental systems based on dimers and trimers of **Br***para***I***meta*-TP and **I***para***Br***meta*-TP molecules on Cu(111), we use in our DFT simulations smaller size halobenzene molecules (i.e. bromobenzene and iodobenzene). These molecules adsorbed on a Cu(111) slab displaying a triangular arrangement are suitable models to accurately reproduce the experimental observations of **Br***para***I***meta*-TP and **Br***para***I***meta*-TP/**I***para***Br***meta*-TP trimers. In particular, we study two windmill structures on Cu(111): One formed by three bromobenzene molecules and another constituted by two bromobenzene molecules plus one iodobenzene molecule. In both cases halogen atoms prefer to occupy bridge positions while the benzene rings stay on fcc-hollow positions. We checked that this configuration is more energetically favorable than other highly symmetrical choices, in agreement with previous calculations of single-molecule adsorption [2].

These structures were fully relaxed following a conjugate gradient algorithm until forces upon atoms were smaller than 0.01 eV$Å^{-1}$ while each electronic self-consistent loop was calculated with a precision of 10^{-5} eV. The copper substrate was modeled with a rectangular three-layer slab of 21.80 Å × 17.62 Å, $5\sqrt{3} \times 7,$ 70 atoms per layer). The atoms of the deepest layer were kept fixed in their bulk positions while those of the two uppermost layers were allowed to relax together with all the atoms belonging to the molecules. A vertical vacuum region slightly larger than 23 Å was established between periodical images and a dipole correction along the *z*-axis was also used. The reciprocal space was sampled with a 3 × 3 × 1 Monkhorst-Pack mesh [3] during the structural minimization. Similar optimization procedures were carried out for the gas-phase systems and the single molecules adsorbed on Cu(111).

We get adsorption energies of 1.13 eV and 1.32 eV for the bromobenzene and iodobenzene respectively. The bromine atom is farther from the surface than the rest of the molecule while iodine is shifted down, see Figures S8 and S9. The formation energy of the Br···Br···Br (Br···Br···I) windmill on the gas phase is -0.199 eV (-0.217 eV), while for the windmill on the Cu(111) we obtain -3.493 eV (-3.654 eV).

We approximated the intermolecular interaction of the windmill on the surface by subtracting the adsorption energy of each molecule to the formation energy of the structure (see Table S1). Furthermore, we calculated two of the contributions involved in the reduction of the intermolecular interaction induced by the adsorption. The energy penalty to move the molecules from its optimal adsorption configuration, ΔE(mol-subs), and the intermolecular energy variation, ΔE_{rel}(mol-mol), due to the formation of the windmill with the atomic positions of the molecules on the surface instead of their optimal configuration on the gas phase. With this energy we would like to quantify both how much intermolecular energy is lost due to shift of the iodine atom out of the molecular plane, and the energy lost due to the adjustment of the intermolecular distance of the windmill on the surface, slightly different from the result on the gas phase. We have calculated them by subtracting the energy of the gas phase windmill in their optimal atomic configuration from the energy of windmill, without the surface, but in the atomic configuration of the adsorbed one. As shown in Table S1 both contributions cannot explain the intermolecular energy change suffered by the molecules upon absorption on Cu(111).

Intermolecular Energies (meV)	Br···Br···Br	Br···Br···I
Windmill gas phase	-199	-217
Windmill on Cu(111)	-100	-70
ΔE(mol-subs)	3	6
ΔE_{rel}(mol-mol)	0	7

Table S1. Intermolecular energies of Br···Br···Br and Br···Br···I clusters in gas phase and on Cu(111) and energy contributions to the weakness of the intermolecular energy induced by the Cu: ΔE(mol-subs) is the molecular-substrate energy variation for forming the windmill on the Cu and ΔE_{rel}(mol-mol) shows the intermolecular energy variation due to the atomic displacements on molecules induced by the Cu. These contributions cannot explain the large reduction calculated on the intermolecular energy.

From the DFT calculations described before, we use our recently developed method to simulate the HR-AFM images. A detailed description of this method can be found in our previous work.[4-6] It is based on a total potential $V(R_{\mathrm{tip}})$, constituted by four different contributions: electrostatic V_{ES}, short range V_{SR}, van der Waals V_{vdW} and tilt component V_{tilt}. Essentially, the model describes the electrostatic and short-range interactions in terms of two physical inputs: the total charge densities of the tip and the sample (ρ^{tip} and ρ^{sam}), and the electrostatic potential of the sample ϕ^{sam} which are obtained from a previous plane-wave calculation of the system without the tip. These contributions are given by the following expressions:

$$V_{\mathrm{ES}} = \int \rho^{\mathrm{tip}}(\boldsymbol{r}, R_{\mathrm{tip}})\phi^{\mathrm{sam}}(\boldsymbol{r})d\boldsymbol{r}, \quad (1)$$

$$V_{\mathrm{SR}} = V_0 \int \left[\rho^{\mathrm{tip}}(\boldsymbol{r}, R_{\mathrm{tip}})\rho^{\mathrm{sam}}(\boldsymbol{r})\right]^{\alpha} d\boldsymbol{r}, \quad (2)$$

where α and V_0 are parameters that are obtained by a fitting procedure of force curves for each specific system [5,6]. In our particular case $\alpha = 1.1$ and V_0=26.84 [eV] (Br···Br···Br) or V_0=32.67 [eV] (I···Br···Br). On the other hand, the van der Waals contribution can be directly extracted from the D3 estimation provided in the DFT calculation. Finally, the tilt potential, which accounts for the possible tip's orientations, is given by

$$V_{\mathrm{tilt}} = \frac{1}{2}k'(\Delta x^2 + \Delta y^2), \quad (3)$$

where k' is a spring constant that can be adjusted to improve the matching with the experimental images. In our case we have set $k' = 0.46\ \mathrm{Jm}^{-2}$. Notice that, instead of performing a direct minimization of the CO tilting taking into account the orientation change of the whole CO charge density as done in previous works [4-6], we have proceed with a more efficient approximation valid for small angles. Similarly to P. Hapala, *et al.* [7,8], we minimize the total energy for small displacements in the xy around the initial position of the apex, considering a rigid tip and restricting the movement to the original plane. This approach speeds up the simulations an order of magnitude with small accuracy loss. All AFM images are calculated without including the substrate explicitly because molecules are physisorbed showing minor

discrepancies in the charge density and electrostatic potential when the Cu(111) slab is taken into account. We have chosen the simulated force maps to compare with the experimental AFM images instead of the gradient force images as, with the typical amplitudes used on the experiments, non-negligible variations on the gradient with respect to the tip-sample distance are observed at the distances run by the tip in one oscillation cycle.

Synthesis of Br*para*I*meta*-TP (the procedure has been reported before in Ref. [9])

General:

The chemicals were used as purchased from Sigma-Aldrich, Acros Organics, Alfa Aesar and TCI Europe. Anhydrous solvents were purchased from Acros Organics or obtained from a MBRAUN solvent purification system MB-SPS-800. Products were purified on a Büchi Sepacore® flash chromatography system X-50 (medium pressure liquid chromatography (MPLC)) with Sepacore® Flash cartridges (particle size: 40 – 63 µm). NMR spectra were measured on a Bruker Avance II 200 MHz, Avance II 400 MHz or Avance III 600 MHz spectrometer. The ^{1}H (7.26 ppm) respectively ^{13}C (77.16 ppm) chemical shift of $CDCl_3$ was used as reference. EI-MS spectra were measured on a Finnigan MAT 95 or a GC-MS HP 5890 with a HP 5971 mass detector. Elemental analyses (EA) were measured on a Thermo FlashEA-1112.

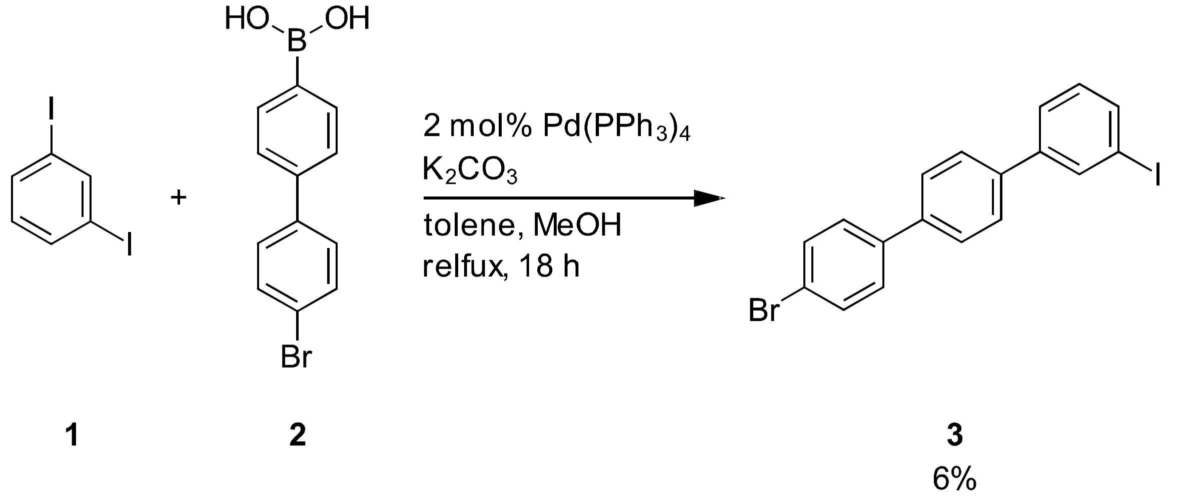

Figure S10. Synthesis of 4-Bromo-3"iodo-p-terphenyl.

Reaction was carried out under an atmosphere of nitrogen, but without dried glassware. No dry solvents were used.

A solution of toluene (20 mL) and methanol (10 mL) was purged by bubbling nitrogen through it for 30 min. 1,3-Diiodobenzene (**1**) (1.01 g, 3.00 mmol, 2.00 equiv.), (4'-bromo-1,1'-biphenyl-4-yl)boronic acid (**2**)[1] (415 mg, 1.50 mmol, 1.00 equiv.), K_2CO_3 (624 mg, 4.51 mmol, 3.01 equiv.) and $Pd(PPh_3)_4$ (35.0 mg, 30.3 µmol, 2.02 mol%) were added. The yellow suspension was stirred under reflux for 18 h. It was quenched with water (40 mL) and diluted with toluene (50 mL). The white precipitate was filtered off. The layers were separated and the organic one was washed with brine (2 x 50 mL), dried over Na_2SO_4, filtered and concentrated under reduced pressure. A white solid was obtained. It was triturated with boiling cyclohexane. The cyclohexane fraction was concentrated. The in this way obtained, white solid was purified by medium pressure liquid chromatography (MPLC) (Silica-gel: 12 g, *n*-hexane/cyclohexane, gradient: 0% to 100%) A white solid was obtained (48.2 mg). It was recrystallized from *n*-hexane. Fine white needles were obtained (42.3 mg, 97.2 µmol, 6%).

^{1}H-NMR (400 MHz, $CDCl_3$, TMS) δ 7.98 (dd, J = 1.8 Hz, 1H), 7.70 (ddd, J = 7.9, 1.3 Hz, 1H), 7.63 (s, 4H), 7.61 – 7.56 (m, 3H), 7.52 – 7.47 (m, 2H), 7.19 (dd, J = 7.8 Hz, 1H).

^{13}C-NMR (101 MHz, $CDCl_3$, TMS) δ 142.90, 139.61, 139.52, 139.03, 136.49, 136.18, 132.12, 130.64, 128.77, 127.74, 127.53, 126.42, 121.94, 95.02.

MS (EI) m/z: 434 (100%); 435 (20%); 436 (97%); 437 (19%).

HR-MS (EI) calc. for $[C_{18}H_{12}BrI]^+$: 433.9167; found: 433.9173.

EA (%) calc. for $C_{18}H_{12}BrI$: C 49.69, H 2.78; found C 49.79, H: 2.74.

(4'-Bromo-1,1'-biphenyl-4-yl)boronic acid was synthesized according to literature. [10]

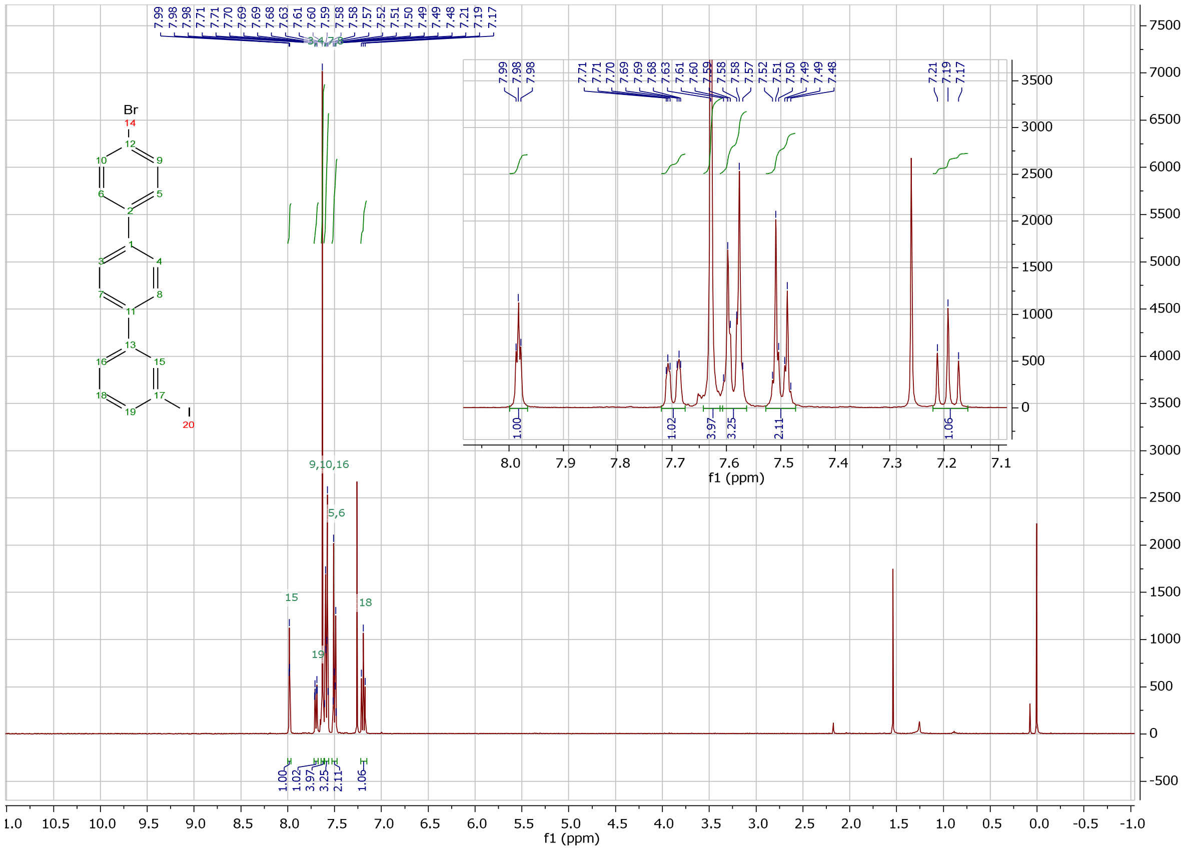

Figure S11: ^{1}H-NMR: 4-Bromo-3''iodo-*p*-terphenyl (**3**) (assignment was carried out with 2D-NMR-spectra)

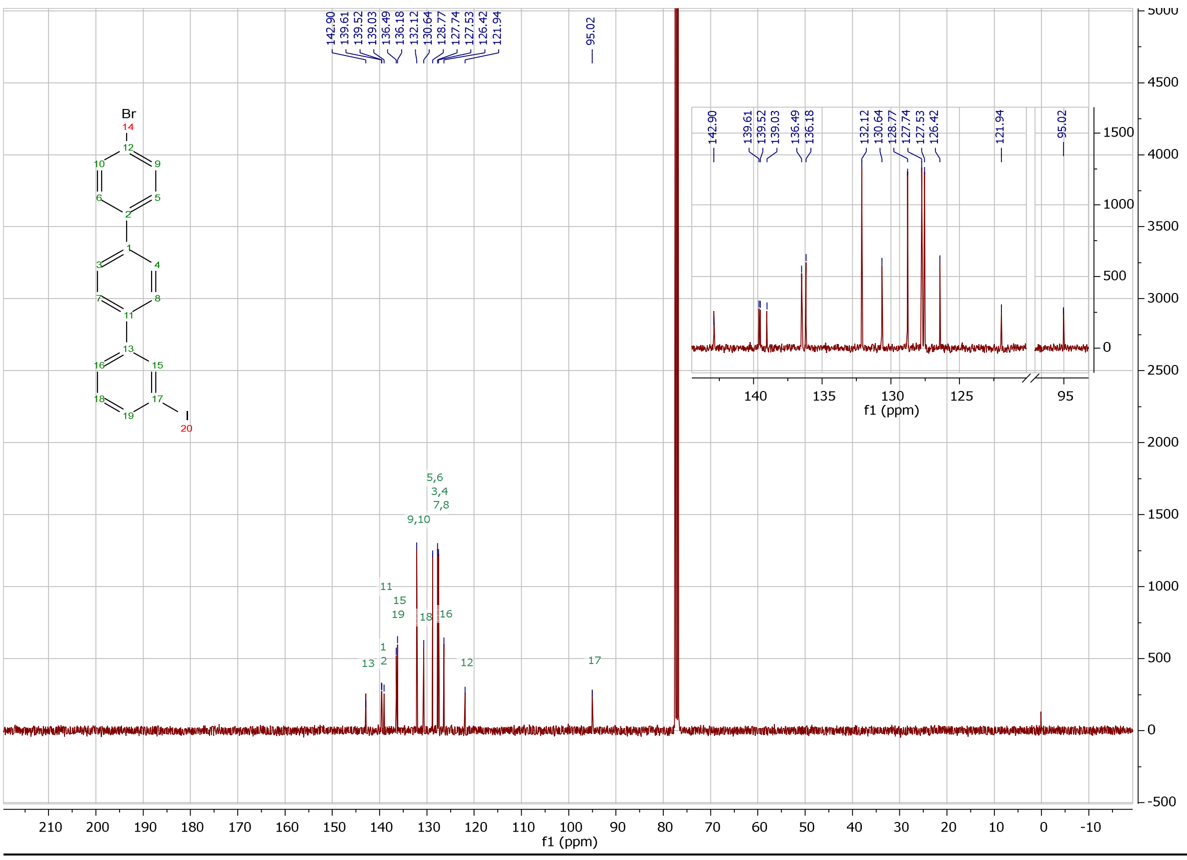

Figure S12: ^{13}C-NMR: 4-Bromo-3"iodo-*p*-terphenyl (**3**) (assignment was carried out with 2D-NMR-spectra):

Synthesis of I*para*Br*meta*-TP

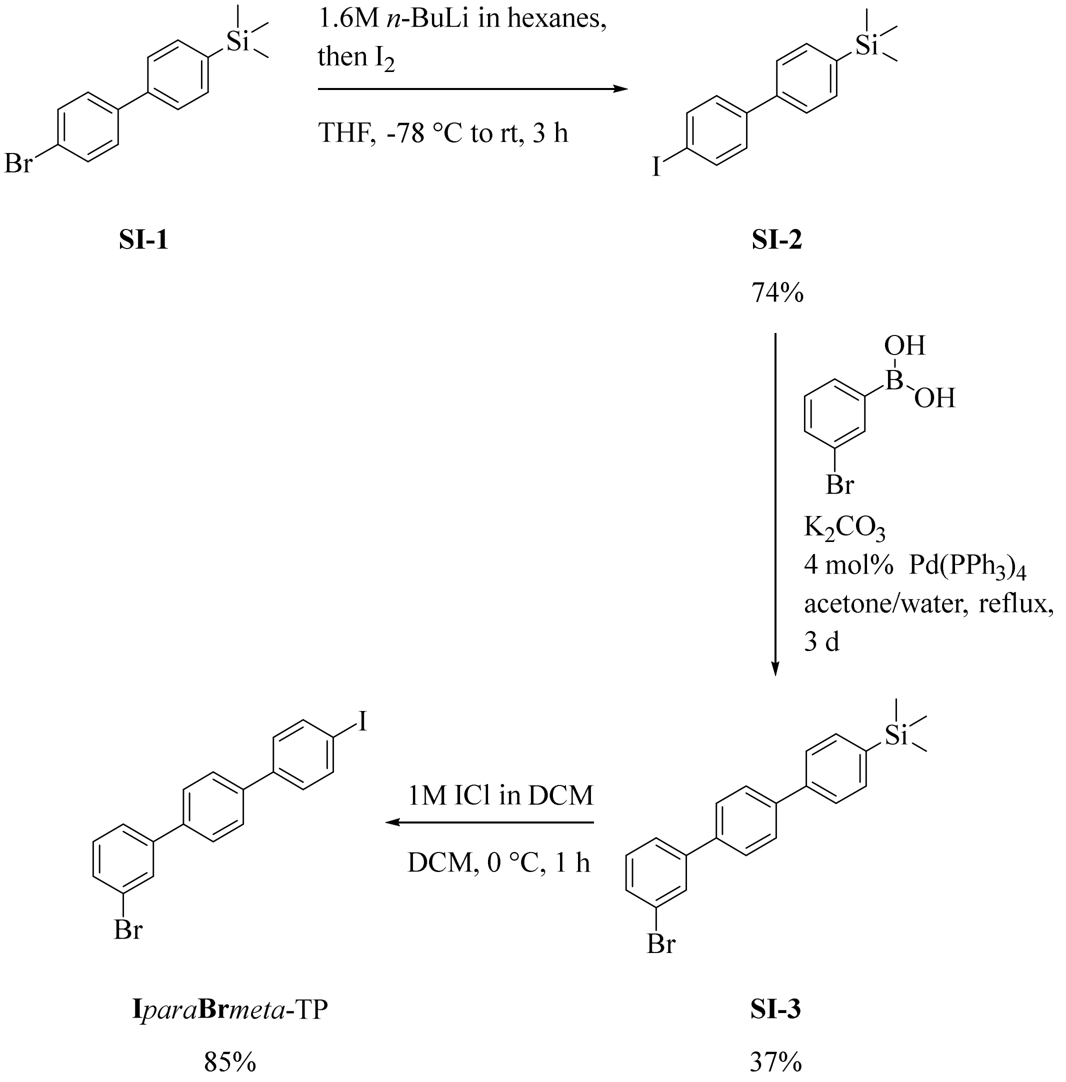

Figure S13. Synthesis of 4-Iodo-3‘‘-bromo-p-terphenyl (I*para*Br*meta*-TP).

General Information for Synthesis:

Reactions involving air and/or moisture sensitive compounds were performed using standard Schlenk techniques under nitrogen.

Chemicals:

The chemicals were purchased from Sigma-Aldrich, Acros Organics, Alfa Aesar and TCI Europe. Anhydrous solvents were purchased from Acros Organics. Deuterated solvents were purchased from Euriso -Top GmbH. Solids were dried over Sicapent® and under high vacuum if necessary. Technical grade solvents, used during work-up and purification, were distilled prior to use. 4-Bromo-4’-(trimethylsilyl)-biphenyl (SI-1) was synthesized according to literature. [11]

NMR:

NMR spectra were measured on a Avance II 400 MHz, Avance III 400 MHz HD or Avance III 600 MHz spectrometer at 25°C. The ^{1}H (7.26ppm) or ^{13}C (77.16ppm) chemical shift of internal residual $CHCl_3$ from $CDCl_3$ was used as reference.

MS:

EI-MS spectra were measured on GC-MS HP 5890 with a HP 5971 mass detector. APCI-MS spectra were measured on a Bruker Micro Mikro-TOF

Elemental Analysis:

Elemental analysis was performed on a CHN-Analysator: Thermo FlashEA - 1112 Series. Samples were weighed on a Mettler Toledo UMX-2 balance.

Column Chromatography:

Flash column chromatography was carried out with Silica 60 M (0.04–0.063mm) from Macherey-Nagel GmbH&Co. KG. Thin layer chromatography was carried out on Polygram®SIL G/UV254 from Macherey-Nagel GmbH&Co. KG.

4-Iodo-4'-(trimethylsilyl)-biphenyl (**SI-2**):

A solution of 4-bromo-4'-(trimethylsilyl)-biphenyl (**SI-1**) [11] (1.22 g, 4.00 mmol, 1.00 equiv.) in THF (dry) (50 mL) was cooled to -78 °C. Then, *n*-butyllithium solution (1.6 M in hexanes, 2.75 mL, 4.40 mmol, 1.10 equiv.) was added drop wise. The pale green solution was stirred for 45 min. The reaction was monitored by GC-MS (EI) analysis of aliquots quenched with water. After complete lithium halogen exchange, iodine (1.22 g, 4.77 mmol, 1.19 equiv.) was added at -78 °C. The reaction mixture was stirred for 1 h at -78 °C. Afterwards the red suspension was allowed to warm to room temperature. It was poured into a sat. aq. Na_2SO_3 solution (50 mL) to quench the reaction. It was diluted with EtOAc (50 mL) and the layers were separated. The organic one was washed with sat. aq. Na_2SO_3 solution (3×25 mL) until it was almost colourless. The combined aqueous fractions was extracted with EtOAc (2×50 mL). Finally, all the combined organic fractions were washed with water (2×50 mL), dried over $MgSO_4$, filtered and concentrated under reduced pressure. A pale red solid was obtained. It was purified by flash column chromatography (SiO_2: 65 g, cyclohexane). A white solid was obtained (1.04 g, 2.96 mmol, 74%). Trace impurities of 4-4''-diiodo-biphenyl were detected. The compound was used without further purification.

^{1}H NMR (400 MHz, chloroform-d) δ 7.77 (d, J = 8.5 Hz, 2H), 7.60 (d, J = 8.2 Hz, 2H), 7.54 (d, J = 8.0 Hz, 2H), 7.34 (d, J = 8.4 Hz, 2H), 0.30 (s, 9H).

3-Bromo-4''-(trimethylsilyl)-p-terphenyl (**SI-3**):

A white suspension of 4-iodo-4'-(trimethylsilyl)-biphenyl (**SI-2**) (845 mg, 2.40 mmol, 1.00 equiv.), 3-bromophenylboronic acid (482 mg, 2.40 mmol, 1.00 equiv.) in acetone (20 mL) and aq. K_2CO_3 solution (2 M, 4.50 mL, 9.00 mmol, 3.75 equiv.) was degassed by bubbling a stream of nitrogen through it for 30 min. Then, $Pd(PPh_3)_4$ (112 mg, 96.0 µmol, 4.00 mol%) was added. The yellow suspension was stirred under reflux for 3 d. It turned from a pale yellow solution into a red suspension (white solid). Afterwards the reaction mixture was cooled to rt

and was diluted with water (25 mL). It was extracted with DCM (3×25 mL). The combined organic fractions were washed with water (25 mL), dried over Na_2SO_4, filtered and concentrated under reduced pressure. A dark red solid was obtained. It was purified by flash column chromatography (SiO2: 115 g, cyclohexane). The target compound was obtained as white solid (354 mg, 0.929 mmol, 37%) and used directly for the next step.

^{1}H NMR (400 MHz, chloroform-d) δ 7.80 (dd, *J* = 1.8 Hz, 1H), 7.73 – 7.61 (m, 8H), 7.57 (d, *J* = 7.8 Hz, 1H), 7.49 (d, *J* = 7.9 Hz, 1H), 7.33 (dd, *J* = 7.8 Hz, 1H), 0.33 (s, 9H).

4-Iodo-3‘‘-bromo-p-terphenyl (**I***para***Br***meta*-TP):

A solution of 3-bromo-4‘‘-(trimethylsilyl)-p-terphenyl (**SI-3**) (100 mg, 262 µmol, 1.00 equiv.) in DCM (dry) (5 mL) was cooled to 0 °C. Then, ICl solution (1 M in DCM, 0.55 mL, 550 µmol, 2.10 equiv.) was added dropwise. After 1 h additional DCM (dry) (5 mL) was added to dissolve a white precipitate that had formed. Then, sat. aq. Na_2SO_3 solution (10 mL) was added and the reaction mixture was stirred until it completely decolorized. Additional DCM was added until all precipitate was dissolved. The layers were separated and the aqueous one was extracted with DCM (3×10 mL). The combined organic fractions were washed with water (20 mL), dried over Na_2SO_4, filtered and concentrated under reduced pressure. A crystalline white solid was obtained. It was recrystallized from *n*-hexane. Plate-shaped colourless crystals were obtained (97.1 mg, 223 µmol, 85%).

^{1}H NMR (600 MHz, chloroform-d) δ 7.80 – 7.77 (m, 3H), 7.64 (s, 4H), 7.55 (ddd, *J* = 7.7, 1.4 Hz, 1H), 7.49 (ddd, *J* = 8.0, 1.9, 1.0 Hz, 1H), 7.39 – 7.36 (m, 2H), 7.33 (dd, *J* = 7.8 Hz, 1H).

^{13}C NMR (151 MHz, dhloroform-d) δ 142.66, 139.98, 139.58, 139.05, 137.97, 130.40, 130.37, 130.09, 128.88, 127.63, 127.37, 125.65, 123.01, 93.31.

HRMS (APCI) m/z [M]+ calc. for $C18H12BrI^+$: 433.9167; found 433.9177.

Elemental Analysis: theo.: %C (49.69), %H (2.78).

Found: %C (49.67), %H (2.58).

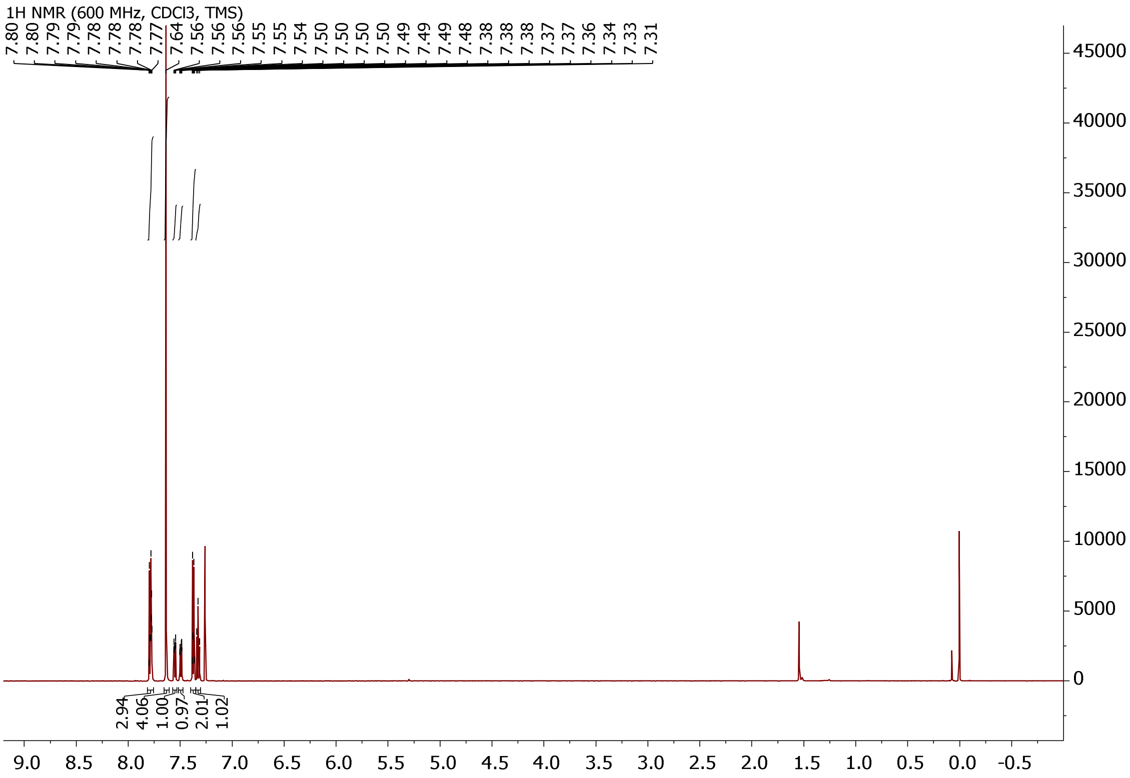

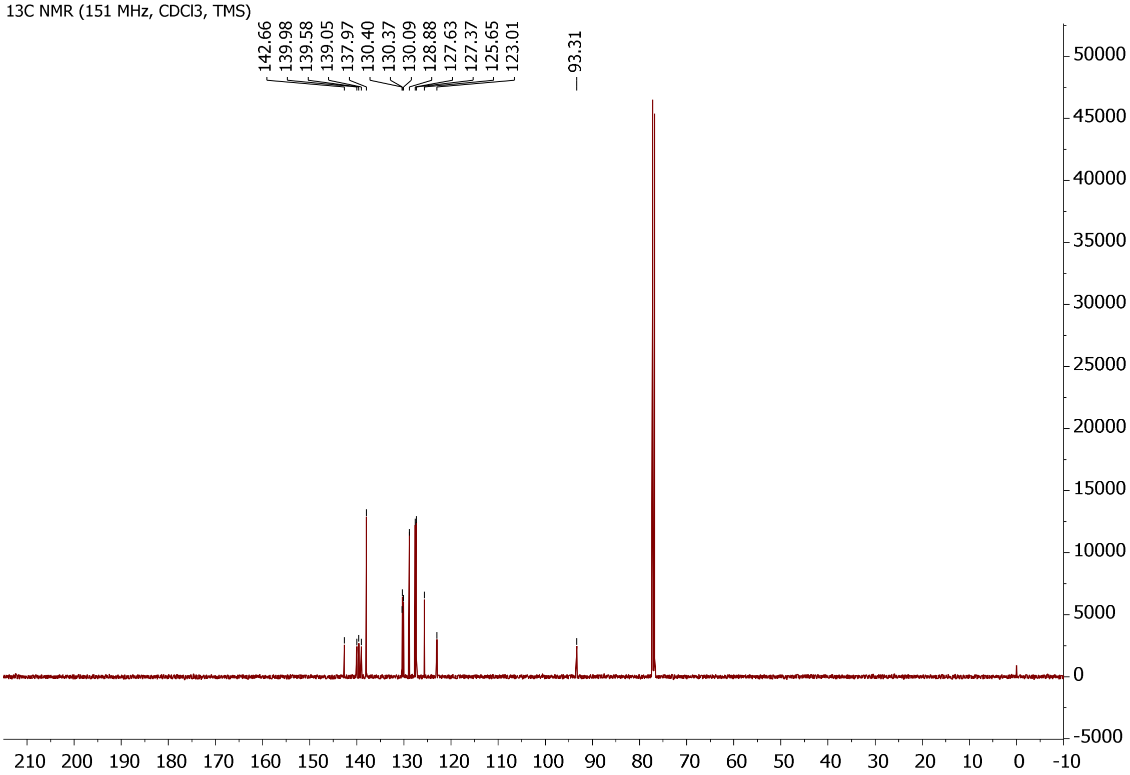

Figure S14. ^{1}H and ^{13}C NMR spectra of I*para*Br*meta*-TP.

References

1 Ebeling, D. *et al.* Adsorption Structure of Mono- and Diradicals on a Cu(111) Surface: Chemoselective Dehalogenation of 4-Bromo-3"-iodo-. *ACS Nano* **13**, 324-336 (2019).

2 Björk, J., Hanke, F. & Stafström, S. Mechanisms of Halogen-Based Covalent Self-Assembly on Metal Surfaces. *J. Am. Chem. Soc.* **135**, 5768-5775 (2013).

3 Monkhorst, H. J. & Pack, J. D. Special points for Brillouin-zone integrations. *Phys. Rev. B* **13**, 5188-5192 (1976).

4 Ellner, M. *et al.* The Electric Field of Co Tips and Its Relevance for Atomic Force Microscopy. *Nano Lett.* **16**, 1974-1980 (2016).

5 Ellner, M., Pou, P. & Pérez, R. Atomic force microscopy contrast with CO functionalized tips in hydrogen-bonded molecular layers: Does the real tip charge distribution play a role? *Phys. Rev. B* **96**, 075418-075418 (2017).

6 Ellner, M., Pou, P. & Pérez, R. Molecular Identification, Bond Order Discrimination, and Apparent Intermolecular Features in Atomic Force Microscopy Studied with a Charge Density Based Method. *ACS Nano* **13**, 786-795 (2019).

7 Hapala, P. *et al.* Mechanism of High-Resolution STM/AFM Imaging with Functionalized Tips. *Phys. Rev. B* **90**, 085421-085421 (2014).

8 Hapala, P., Temirov, R., Tautz, F. S. & Jelínek, P. Origin of High-Resolution IETS-STM Images of Organic Molecules with Functionalized Tips. *Phys. Rev. Lett.* **113**, 226101-226101 (2014).

9 Ebeling, D. *et al.* Chemical bond imaging using higher eigenmodes of tuning fork sensors in atomic force microscopy. *Appl. Phys. Lett.* **110**, 183102, doi:10.1063/1.4982801 (2017).

10 Galda, P. & Rehahn, M. A Versatile Palladium-Catalyzed Synthesis of n-Alkyl-Substituted Oligo-p-phenyls. *Synthesis* **1996**, 614-620, doi:10.1055/s-1996-4260 (1996).

11 Hanss, D. & Wenger, O. S. Conformational Effects on Long-Range Electron Transfer: Comparison of Oligo-p-phenylene and Oligo-p-xylene Bridges. *Eur. J. Inorg. Chem.* **2009**, 3778-3790, doi:10.1002/ejic.200900396 (2009).
